# Inequalities in the care for Labor and Delivery in Rio de Janeiro – Birth in Brazil Research II: national survey on abortion, delivery, and birth

**DOI:** 10.11606/s1518-8787.2025059006516

**Published:** 2025-10-20

**Authors:** Maria do Carmo Leal, Ana Paula Esteves-Pereira, Rosa Maria Soares Madeira Domingues, Sonia Duarte de Azevedo Bittencourt, Mariza Miranda Theme-Filha, Neide Pires Leal, Marcos Nakamura-Pereira, Marcos Augusto Bastos Dias, Thaiza Dutra Gomes de Carvalho, Tatiana Henriques Leite, Silvana Granado Nogueira da Gama

**Affiliations:** I Fundação Oswaldo Cruz. Escola Nacional de Saúde Pública. Departamento de Métodos Quantitativos em Saúde. Rio de Janeiro, RJ, Brasil; II Fundação Oswaldo Cruz. Instituto Nacional de Infectologia Evandro Chagas. Laboratório de Pesquisa Clínica em DST/Aids. Rio de Janeiro, RJ, Brasil; III Fundação Oswaldo Cruz. Instituto Nacional da Saúde da Mulher da Criança e do Adolescente Fernandes Figueira. Rio de Janeiro, RJ, Brasil; IV Universidade do Estado do Rio de Janeiro. Instituto de Medicina Social. Departamento de Epidemiologia. Rio de Janeiro, RJ, Brasil

**Keywords:** Parturition, Labor, Obstetric, Public sector, Private Sector, Brazil

## Abstract

**OBJECTIVE:**

To describe the care for labor and delivery in the state of Rio de Janeiro, Brazil, according to hospital location and type of funding for delivery, and to verify the social, geographic, and care factors associated with going into labor and having a vaginal delivery.

**METHODS:**

This is a cross-sectional hospital-based study ("Birth in Brazil Research II: national survey on abortion, delivery, and birth") conducted in 29 hospitals located in the state of Rio de Janeiro. Women with live births and/or stillbirths with gestational age ≥ 22 weeks or ≥ 500 g weight were eligible, totaling 1,762 women. Interviews were conducted in the hospitals, in the immediate postpartum period. Data were extracted from the prenatal card and maternal medical records. Multiple logistic regression was performed for labor and delivery, using a hierarchical model, with estimated odds ratios and specific confidence intervals.

**RESULTS:**

The frequency of going into labor was 54% and of vaginal delivery, 41.0%. The following aspects were associated with going into labor: provision of care in hospitals located in the municipality of Rio de Janeiro, with public source of funding, being nulliparous or multiparous with previous delivery, preferring vaginal delivery at the end of pregnancy, not being obese and without complications during pregnancy. For vaginal delivery, we observed an association with low level of education, having no partner, being nulliparous or multiparous with previous delivery, having access to good practices as for going into labor and delivery, and use of analgesia during labor, regardless of the type of funding and hospital location.

**CONCLUSIONS:**

We observed advances in labor care in the state of Rio de Janeiro, although the frequency of labor and vaginal delivery is still low, as well as good practices, but with better results for the municipality of Rio de Janeiro. All good practices were associated with vaginal delivery, especially the use of analgesia and the presence of doulas. Vaginal delivery was more frequent in socially vulnerable women.

## INTRODUCTION

The state of Rio de Janeiro (RJE) has the third largest population in the country, 16,055,164 inhabitants, according to the 2022 demographic census. It gathers, in its metropolitan region (MR), 75% of the population of the state, of which more than half resides in the municipality of Rio de Janeiro (MRJ)^1^.

The history of the constitution of the RJE was a strong determinant of the characteristics of public health in the state. With the heritage of the period in which it was the capital of Brazil, the MRJ has a structure of public health services very distinct from all other municipalities.

Since the end of the 1990s, the MRJ has developed a humanization policy of childbirth care, promoting good practices in the care for labor and delivery^2^. These measures include reform of the physical space of maternity hospitals, aiming at a more comfortable and welcoming environment; care protocols for the adoption of good practices and abandonment of harmful ones; incorporation of obstetric nursing into labor care; and encouragement of the companion's presence, even before the publication of the federal law^3,4^. Subsequently, in 2011, with the implementation of the *Cegonha Carioca* program (a program by the government of Rio de Janeiro aimed at providing and guaranteeing a humanized, top-notch care for mothers and babies), the whole network of reference of maternity hospitals for the care for labor of pregnant women with prenatal care (PC) in public units was defined, with guarantee of vacancy and transport from the house to the hospital at the time of delivery^5,6^.

Twelve years ago, authors of the study *Nascer no Brasil* (Birth in Brazil – NB) already identified that obstetric care in the country was interventionist, without respecting the physiology of delivery or prioritizing the autonomy of women, in contrast to the recommendations of the World Health Organization (WHO)^7^ and the Brazilian Ministry of Health (MS)^8^, which were based on scientific evidence, already available at that time, on the promotion of good practices and abandonment of unnecessary interventions^9^.

The hypothesis of this article is that the MRJ presents a higher frequency of good practices in the care for labor and delivery compared to the other regions of the RJE.

Hence, in this study our objective was:

- To describe the care for labor in the RJE according to the location of the hospital (MRJ; other municipalities of the MR; and small cities of the state) and the source of funding for delivery;
- To verify social, geographic, and care inequalities and their association with going into labor and having a vaginal delivery (VD).

## METHODS

### Birth in Brazil Research II

A cross-sectional hospital-based study, with national coverage and two follow-up waves (from Portuguese, *Nascer no Brasil II: pesquisa nacional sobre aborto, parto e nascimento* [Birth in Brazil II: national survey on abortion, delivery, and birth] – NBII)", in the period 2021–2024. The protocol of the aforementioned study is published in Leal et al.^10^.

### Study in the State of Rio de Janeiro

#### Design and Location

A cross-sectional hospital-based study was conducted in 29 hospitals with ≥ 100 live births (LB)/year (according to the Live Birth Information System [*Sistema de Informação sobre Nascidos Vivos* – SINASC], 2017), located in 18 municipalities of the RJE.

#### Population and Period

Women admitted to delivery and their newborns between November 2021 and June 2023.

#### Sample Calculation

The sample was calculated based on the proportion of cesarean sections in the RJE in 2019 (57%), 5% significance level, and 90% power, to detect differences of 7%. A design effect of 1.3 was used, resulting in the minimum sample size of 1,350 women admitted to delivery. According to *post-hoc* calculations, the sample has the power to detect absolute differences of 5% for outcomes with prevalence between 5 and 20%.

#### Sample Design

A two-stage probability sampling was selected:

- Stage 1: Hospitals were stratified by location (MRJ, MR, and small cities), type (public, mixed, private), and size (100–499, ≥ 500 LB/year). Of the total of 120 hospitals with ≥ 100 LB/year in the RJE (according to SINASC–2017), 29 were drawn for the research;
- Stage 2: Women were identified by hospital records (census and hospitalization book), which were then registered in a single list, organized in chronological order by date of delivery. The selection of participants was performed consecutively until reaching the stipulated sample number: 30 women in hospitals with 100–499 LB/year; 50 women in private hospitals with ≥ 500 LB/year; and 90 women in public and mixed hospitals with ≥ 500 LB/year.

#### Selection Criteria

Puerperae with delivery of LB with any gestational weight or age, and mothers of stillbirths with gestational age ≥ 22 weeks or weight ≥ 500 g were included. Women with triplet or multiple delivery were excluded, those with communication difficulties (foreign, Indigenous women who did not understand Portuguese, people with hearing impairment or severe mental illness), and those hospitalized for delivery by court order. In total, 1,762 women admitted to delivery were interviewed, being 1,752 LB and ten stillbirths.

Face-to-face interviews were conducted in the immediate puerperium, and PC cards were photographed, when available, and clinical data were extracted from the hospital medical record.

#### Outcome Variables

Two outcome variables were analyzed:

- Going into labor;
- Having a VD.

All women whose labor spontaneously started or who had induced labor, who reached cervical dilation ≥ 4 cm, were classified as having gone into labor.

For the VD outcome, all women with VD were considered, including those with the use of forceps or vacuum extractor.

#### Exposure Variables for the Outcome "Going into Labor"

Geographic location of the hospital (MRJ, MR, rural towns); source of funding for delivery (public, private); self-reported skin color/race (white, Black, mixed-race, Asian, Indigenous, categories used by the Brazilian Institute of Geography and Statistics); age (< 20, 20–34, ≥ 35 years); years of formal study (≤ 7, 8–10, 11–14, ≥ 15); lives with a partner (no, yes); obstetric history (nulliparous, multiparous with vaginal delivery only, multiparous with cesarean section); PC adequacy (no, yes); type of delivery preference at the end of pregnancy (vaginal, cesarean section, no preference); has sought more than one maternity hospital for delivery — traveling (no, yes); obesity (body mass index — BMI < 30, BMI ≥ 30); obstetric risk (no, yes); time of admission to delivery.

Women with delivery in public or private hospitals linked to the Brazilian Unified Health System (SUS) without payment of hospitalization by health insurance were classified as having "public source of funding"; those with delivery in private hospitals and payment by health insurance or direct disbursement, as having "private source of funding."

For the classification of PC adequacy, the onset up to 12 weeks of gestation and the number of visits according to gestational age (GA) was used, according to the following schedule: GA ≥ 20 and < 26 = visits ≥ 2; GA ≥ 26 and < 30 = visits ≥ 3; GA ≥ 30 and < 34 = visits ≥ 4; GA ≥ 34 and < 36 = visits ≥ 5; GA ≥ 36 and < 38 = visits ≥ 6; GA ≥ 38 and < 40 = visits ≥ 7; GA ≤ 40 = visits ≥ 8^3^.

The obstetric risk criterion was defined as the presence of some of the following conditions: twin pregnancy, chronic arterial hypertension (AH); gestational AH; preeclampsia; eclampsia; HELLP syndrome (severe preeclampsia with hemolysis, elevated liver enzymes, low platelet count), pregestational diabetes; gestational diabetes; severe chronic disease (lupus, scleroderma, kidney disease, heart disease, cancer); infections upon hospital admission; premature placental abruption (PPA); placenta accreta/increta/percreta and intrauterine growth restriction (IUGR); and hospitalization due to any reason during the current pregnancy.

For calculating the time between hospital admission and delivery, the interval between the date and time of hospital admission and the date and time of delivery was considered, with subsequent categorization in quintiles: 1st quintile (< 2 h), 2nd quintile (2 h to < 5 h), 3rd quintile (5 h to < 8 h), 4th quintile (8 h to < 16h), and 5th quintile (≥ 16 h). Women from the first and last quintile were excluded from the analyses of crude and adjusted regressions. The former did not have enough time to receive good practices and the latter presented a prolonged period, suggesting the presence of complications that could interfere with adherence to good practices.

#### Exposure Variables for the Outcome "Vaginal Delivery"

All covariates previously described were used, and variables related to the good practices of labor were included, that is: presence of companion during labor/delivery, presence of a doula, ingestion of liquids or food during labor, freedom of movement during labor, use of non-pharmacological methods for pain relief, and performance of obstetric nurse/obstetrician (ON/OB) during labor/delivery. The following were considered as interventions during labor: use of peripheral venous catheter, use of oxytocin (hormone used for induction and/or acceleration of labor), amniotomy (artificial rupture of ovular membranes), use of analgesia in labor (epidural or rachidian), lithotomy (dorsal position in childbirth), and Uterine fundal pressure maneuver (external pressure on the woman's uterus).

The information used for the classification of obstetric risk, labor, type of delivery, time between admission and delivery, use of oxytocin, amniotomy, and analgesia in labor were obtained from the hospital medical records. The other variables were obtained during the interview with the puerperae.

#### Data Analysis

Descriptive data analyses were stratified by the location of the hospitals where the parturients were seen and by the source of funding for delivery. The proportions and their 95% confidence intervals (95%CI) were estimated.

A theoretical model was used to classify the explanatory variables according to their hierarchical level in relation to the outcome (distal, intermediate, and proximal).

- Distal variables: age, skin color, years of formal study, lives with a partner, source of funding for delivery;
- Intermediate: obstetric history, PC adequacy, traveling to delivery, obesity, obstetric risk, type of delivery preference at the end of pregnancy;
- Proximal: presence of a companion, doula, ON/OB, feeding, freedom of movement, use of non-pharmacological methods for pain relief, use of peripheral venous access, oxytocin, epidural analgesia, amniotomy, lithotomy, and Uterine fundal pressure maneuver.

For the analysis of the outcome "going into labor," only distal and intermediate variables entered the model. For the analysis of the "vaginal delivery" outcome, variables from the three levels were included, but only women who had assisted birth in the hospital were included in the analysis, and those who had indication of cesarean section at the time of hospital admission were excluded.

At each level, the variables were selected by the criterion of p < 0.20 for remaining in the model. For subsequent analyses, those that presented association (p < 0.05) with the outcomes were maintained in the model, after adjustment by the variables of the same level and higher hierarchical levels. The crude and adjusted odds ratio and 95%CI were calculated.

All analyses were carried out using the IBM SPSS Statistics for Windows software, version 23.0 (IBM Corp. Armonk, NY, USA), including the design effect, weighting, and calibration of the sample. For calibration, groups formed by the combination of stratum, type of delivery (vaginal or cesarean section), and women's age (10–19, 20–34, ≥ 35) were used, having SINASC-2022 as reference.

The NBII research was approved by the National Commission of Ethics in Research (*Comissão Nacional de Ética em Pesquisa* – CONEP), with Certificate of Presentation for Ethical Consideration (CAAE): 21633519.5.0000.5240, on March 11, 2020, and, in its absence, it was approved by the local research ethics committees of the institutions or the clinical board's.

## RESULTS

In the RJE, deliveries are concentrated in the MRJ and other municipalities of the MR, with a fourth taking place in small cities, with 74% having public source of funding. Most women are mixed-race as for skin color/race, are 20 to 34 years old, have 12 or more years of formal study, and live with a partner. Most of them have previous deliveries, start PC up to 12 gestational weeks, and have adequate PC, with 12% reporting traveling for delivery (Table 1). One third of the women presented some obstetric risk and one fourth presented obesity. Approximately 50% went into labor, with 41% having VD and 71%, the presence of a companion. Most deliveries occurred until the first 8 hours of hospitalization. As for women going into labor, more than 60% were accompanied and were assisted by nurses, but less than 50% were fed, had freedom of movement, and used non-pharmacological methods. The most frequent intervention was the use of venous access (48%), with less than 15% of women using oxytocin, analgesia, and amniotomy. Almost all women delivered in vertical positions, 17% with ON assistance, and 7% were submitted to Uterine fundal pressure maneuver and episiotomy (Table 2).

**Table 1:** Sociodemographic characteristics and those related to obstetric history and prenatal care, according to hospital location and type of funding for delivery, Birth in Brazil II, state of Rio de Janeiro (NBII-ERJ), 2021–2023.

		Municipality of Rio de Janeiro	Metropolitan Region	Other Municipalities	Public	Private	TOTAL
		% (CI)	% (CI)	% (CI)	% (CI)	% (CI)	% (CI)
Total postpartum women		711	631	420	1,305	457	1,762
*Sociodemographic*							
Hospital location							
	Municipality of Rio de Janeiro	-	-	-	36.2 (8.3–77.9)	52.1 (26.1–76.9)	40.4 (15.6–71.1)
	Metropolitan Region	-	-	-	36.0 (12.5–68.9)	34.9 (14.8–62.2)	35.8 (16.1–61.7)
	Other Municipalities	-	-	-	27.6 (12.8–49.8)	12.9 (6.7–23.5)	23.8 (12.3–41.1)
Source of funding for delivery							
	Public	66.5 (25.0–92.1)	74.6 (57.1–86.7)	85.9 (78.7–90.9)	-	-	74.0 (61.6–83.5)
	Private	33.4 (7.8–74.9)	25.3 (13.2–42.8)	14.1 (9.7–21.2)	-	-	25.9 (16.4–38.3)
Skin color							
	White	33.1 (23.4–44.5)	24.6 (19.3–30.8)	34.4 (21.7–49.8)	23.3 (19.0–28.3)	50.5 (43.7–74.3)	30.4 (26.0–35.1)
	Black	21.5 (13.5–32.5)	23.0 (19.6–26.7)	15.8 (11.1–22.2)	24.7 (21.2–28.5)	9.3 (6.4–13.1)	20.7 (16.9–25.1)
	Mixed-race	45.4 (40.1–50.7)	52.4 (48.3–56.6)	49.8 (39.8–59.7)	52.0 (46.8–57.2)	40.2 (32.8–48.1)	49.0 (45.2–52.7)
Mother's age (years)							
	10 to 19	10.6 (6.1–18.0)	9.7 (7.0–13.4)	10.4 (7.0–15.3)	13.4 (11.2–15.9)	1.4 (0.5–3.8)	10.3 (7.9–13.2)
	20 to 34	67.4 (63.5–71.0)	74.8 (70.5–78.6)	73.3 (68.6–77.6)	73.4 (70.3–76.3)	65.7 (61.2–69.9)	71.4 (69.2–73.6)
	35 or over	21.9 (14.1–32.2)	15.3 (11.5–20.2)	16.1 (13.8–18.7)	13.1 (11.7–14.6)	32.7 (28.4–37.3)	18.2 (15.9–20.7)
Level of education (years)							
	≤ 8 (some ES)	12.1 (7.0–20.2)	15.2 (11.0–20.5)	17.1 (11.5–24.7)	19.1 (15.9–22.8)	0.9 (0.5–1.7)	14.4 (11.9–17.3)
	9 to 11 (ES)	30.7 (15.6–51.4)	25.2 (20.7–30.4)	29.5 (25.9–33.3)	35.5 (28.2–43.5)	8.4 (5.1–13.6)	28.5 (20.9–37.4)
	12 to 15 (HS)	33.3 (26.9–40.4)	46.9 (42.0–51.8)	44.4 (38.3–50.7)	40.1 (33.2–47.5)	42.8 (34.9–51.1)	40.8 (35.2–46.7)
	≥ 16 (CD)	23.6 (9.3–48.3)	12.5 (6.0–24.2)	8.8 (5.4–13.9)	5.1 (3.5–7.2)	47.7 (36.9–58.7)	16.1 (11.5–22.0)
	Lives with a partner	80.8 (69.9–88.4)	79.4 (71.6–85.4)	83.9 (78.9–87.9)	76.5 (72.6–80.1)	93.9 (89.7–96.5)	81.0 (76.6–84.8)
*Obstetric history*							
Parity							
	Nulliparous	46.8 (39.7–53.9)	42.0 (35.3–49.1)	40.8 (33.2–48.8)	39.4 (35.9–43.0)	55.8 (49.1–62.3)	43.6 (40.2–47.2)
	1 to 2 previous deliveries	43.7 (40.6–46.7)	46.8 (42.0–51.3)	47.5 (41.9–53.2)	47.4 (44.8–50.1)	40.5 (35.1–46.3)	45.6 (43.3–48.0)
	3 or more previous deliveries	9.5 (5.8–15.2)	11.2 (8.7–14.3)	11.5 (9.3–14.2)	13.1 (11.8–14.5)	3.5 (2.0–6.1)	10.6 (9.0–12.4)
History of previous cesarean section							
	Primiparous	46.8 (39.7–53.9)	42.0 (35.3–49.1)	40.8 (33.2–48.8)	39.4 (35.9–43.0)	55.8 (49.1–62.3)	43.6 (40.2–47.2)
	Multiparous with previous cesarean section	24.1 (19.5–29.1)	28.7 (23.9–33.8)	38.7 (33.1–44.5)	27.7 (23.2–32.8)	33.1 (28.5–38.0)	29.1 (25.2–33.4)
	Multiparous with previous vaginal delivery only	29.1 (19.2–41.5)	29.3 (22.6–37.1)	20.5 (14.1–28.8)	32.8 (28.6–37.2)	11.1 (7.5–16.2)	27.1 (22.3–32.6)
*Type of delivery preference at the end of pregnancy*							
	Vaginal	56.7 (44.2–68.5)	55.4 (46.2–64.3)	51.6 (44.8–58.4)	63.8 (59.9–67.5)	30.1 (23.9–37.1)	55.0 (49.9–60.1)
	Cesarean section or without preference	43.3 (31.5–55.8)	44.6 (35.7–53.8)	48.4 (41.6–55.2)	36.2 (32.5–40.1)	69.9 (62.9–76.1)	45.0 (39.9–50.1)
*Prenatal care*							
Trimester of prenatal care onset							
	First trimester (up to 12 weeks)	84.0 (75.7–89.9)	75.6 (70.6–79.9)	79.6 (68.9–87.3)	74.7 (70.6–78.3)	94.9 (92.1–96.7)	80.0 (76.9–82.7)
	Second trimester (13 to 27 weeks)	15.8 (9.9–24.2)	21.9 (18.1–26.3)	17.3 (11.5–25.2)	23.2 (20.7–25.8)	4.7 (3.1–7.1)	18.3 (16.0–20.9)
	Third trimester (≥ 28 weeks)	0.1 (0.0–1.1)	2.3 (1.5–3.6)	2.9 (1.2–6.8)	2.0 (0.9–4.3)	0.3 (0.0–2.5)	1.6 (0.7–3.2)
	Prenatal care adequacy^a^	72.9 (58.8–83.5)	60.9 (54.9–66.6)	67.7 (58.9–75.4)	60.1 (56.4–63.6)	89.7 (84.8–93.1)	67.4 (63.8–70.8)
	Traveling for delivery	5.6 (2.9–10.5)	17.9 (12.6–24.8)	13.8 (4.6–34.7)	15.5 (9.4–24.5)	1.9 (0.9–4.0)	12.0 (7.8–17.8)

CI: confidence interval.

ES: Elementary School.

HS: High School.

CD: College Degree.

a Onset up to 12 weeks of gestation and number of visits appropriate for gestational age at birth according to WHO criteria.

**Table 2:** Characteristics of obstetric risk, type of delivery, good practices and interventions during labor and delivery, according to hospital location and type of funding for delivery, Birth in Brazil II, state of Rio de Janeiro (NBII-ERJ), 2021–2023.

		Municipality of Rio de Janeiro	Metropolitan Region	Other Municipalities	Public	Private	TOTAL
		% (CI)	% (CI)	% (CI)	% (CI)	% (CI)	% (CI)
Total postpartum women		711	631	420	1,305	457	1,762
*Pregestational obesity*		22.1 (18.3–26.4)	25.5 (18.7–33.6)	27.2 (19.9–36.1)	24.6 (19.5–30.6)	24.2 (20.0–29.0)	24.5 (20.5–28.9)
*Obstetric risk*							
	Diabetes or gestational hypertension	17.5 (15.6–19.7)	25.6 (12.6–44.9)	27.8 (19.6–37.7)	24.2 (15.6–35.7)	19.0 (15.0–23.7)	22.9 (16.4–31.0)
	Severe chronic disease	0.6 (0.3–1.4)	0.6 (0.3–1.3)	0.8 (0.2–3.2)	0.7 (0.3–1.3)	0.7 (0.2–2.3)	0.7 (0.4–1.2)
	Infections upon hospital admission	5.3 (2.6–10.6)	1.9 (0.8–4.5)	3.6 (1.4–9.1)	4.7 (2.6–8.5)	0.7 (0.2–2.8)	3.7 (1.9–7.1)
	Premature placental abruption	0.9 (0.5–1.5)	1.5 (0.5–4.5)	0.8 (0.4–1.6)	1.3 (0.7–2.5)	0.5 (0.1–1.7)	1.1 (0.6–2.0)
	Placenta accreta/ increta/percreta	0.4 (0.1–1.8)	0.5 (0.1–1.4)	0.5 (0.1–2.4)	0.4 (0.1–1.2)	0.5 (0.2–1.6)	0.5 (0.2–1.0)
	Intrauterine growth restriction	0.9 (0.2–3.5)	0.7 (0.3–1.8)	2.3 (1.6–3.3)	1.2 (0.6–2.3)	1.2 (0.6–2.6)	1.2 (0.7–2.0)
	Twin pregnancy	1.0 (0.2–4.0)	1.9 (1.0–3.6)	1.1 (0.4–2.8)	1.1 (0.4–2.9)	1.9 (0.8–4.1)	1.3 (0.6–2.7)
	HIV diagnosis	0.2 (0.0–1.4)	1.5 (0.6–4.0)	0.6 (0.2–2.0)	1.0 (0.3–2.8)	0.3 (0.0–1.3)	0.8 (0.3–2.0)
	Hospitalization during pregnancy	2.0 (1.4–2.7)	2.2 (0.9–5.1)	4.7 (1.7–12.2)	3.1 (1.7–5.7)	1.5 (0.8–2.7)	2.7 (1.6–4.5)
	Any of the aforementioned risks	26.8 (24.1–29.6)	31.5 (15.4–53.8)	36.6 (28.6–45.3)	33.2 (23.4–44.6)	24.0 (20.1–28.4)	30.8 (23.5–39.2)
*Labor*							
	Went into labor	63.5 (36.1–84.3)	51.0 (39.9–62.1)	43.9 (34.5–53.6)	66.0 (53.3–76.7)	21.2 (15.2–28.7)	54.4 (41.2–66.9)
	*Type of delivery*						
	Vaginal delivery^a^	46.1 (28.7–64.5)	40.4 (30.6–51.1)	33.4 (23.9–44.4)	50.3 (43.3–57.4)	14.5 (10.3–20.0)	41.0 (32.6–50.0)
	Cesarean section	53.8 (35.4–71.2)	59.5 (48.8–69.3)	66.5 (55.5–76.0)	49.6 (42.5–56.6)	85.4 (79.9–89.6)	58.9 (49.9–67.3)
	Antepartum cesarean section	36.4 (15.6–63.8)	48.9 (37.8–60.0)	56.0 (46.3–65.4)	33.9 (23.2–46.6)	78.7 (71.2–84.7)	45.5 (33.0–58.7)
	Intrapartum cesarean section	17.4 (10.9–26.6)	10.5 (7.9–13.9)	10.5 (6.9–15.5)	15.6 (10.9–22.0)	6.6 (3.8–11.1)	13.3 (9.1–19.0)
	Women going into labor	452	322	185	862	97	959
*Time between hospitalization and delivery (vaginal or intrapartum cesarean section)*							
	1st quintile (< 2 h)	18.9 (16.6–21.4)	20.8 (15.9–26.9)	21.4 (11.1–37.2)	20.1 (16.8–24.0)	18.8 (11.1–30.1)	20.0 (16.9–23.6)
	2nd quintile (2 h to < 5 h)	19.8 (16.8–23.2)	22.2 (17.3–27.9)	24.5 (19.9–29.7)	20.7 (18.1–23.5)	28.9 (18.9–41.4)	21.5 (18.7–24.6)
	3rd quintile (5 h to < 8 h)	20.9 (18.0–24.0)	14.9 (12.5–17.8)	15.1 (10.6–21.0)	17.4 (13.6–21.9)	21.2 (13.0–32.6)	17.8 (14.4–21.7)
	4th quintile (8 h to < 16 h)	20.7 (18.1–23.6)	19.6 (16.6–23.1)	18.7 (15.6–22.2)	19.7 (18.3–21.3)	22.2 (15.0–31.6)	20.0 (18.4–21.6)
	5th quintile (≥ 16 h)	19.7 (17.4–22.3)	22.4 (16.6–29.6)	20.4 (13.2–30.0)	22.1 (19.1–25.3)	8.9 (3.6–20.6)	20.8 (18.1–23.7)
*Good practices*							
	Presence of a companion during labor and delivery	88.8 (83.1–92.8)	50.2 (30.5–69.9)	82.4 (62.9–92.8)	71.9 (53.0–85.3)	98.8 (93.2–99.8)	74.6 (58.7–85.9)
	Presence of a doula	4.3 (3.1–5.9)	0.8 (0.1–4.3)	3.8 (1.5–9.7)	2.3 (1.1–4.5)	4.7 (2.8–8.0)	2.9 (1.9–4.5)
	Was able to feed herself	34.9 (28.5–41.8)	34.0 (22.6–47.7)	34.9 (28.7–41.7)	33.7 (28.4–39.5)	42.6 (33.1–52.6)	34.6 (29.5–40.1)
	Was able to move	53.8 (45.8–61.6)	46.7 (38.8–54.9)	44.1 (28.4–61.0)	43.3 (39.4–47.2)	44.5 (29.3–60.8)	43.4 (39.4–47.4)
	Use of non- pharmacological methods	43.1 (39.5–46.8)	42.5 (36.1–49.1)	45.4 (32.4–59.1)	49.9 (44.5–55.3)	46.7 (33.1–60.8)	49.5 (44.4–54.7)
	Labor accompanied by a nurse	61.1 (53.3–68.4)	61.3 (49.5–71.9)	66.0 (50.4–78.7)	65.9 (59.8–71.5)	38.0 (24.7–53.5)	62.3 (56.4–67.9)
*Interventions*							
	Use of peripheral venous access	47.9 (41.2–54.7)	45.0 (38.6–51.5)	51.9 (45.7–58.0)	45.1 (42.3–48.0)	70.4 (61.5–78.0)	47.7 (44.2–51.3)
	Use of oxytocin	11.9 (6.5–20.6)	14.5 (11.1–18.7)	17.0 (7.8–33.3)	12.5 (8.5–18.1)	24.4 (14.2–38.7)	13.7 (9.5–19.5)
	Use of analgesia	5.3 (1.1–22.2)	3.5 (1.6–7.2)	1.9 (0.7–4.7)	1.2 (0.4–3.0)	29.5 (17.2–45.8)	4.0 (1.9–8.4)
	Amniotomy	15.1 (10.0–22.2)	16.5 (11.6–23.1)	7.4 (5.1–10.7)	12.8 (10.3–15.8)	25.5 (14.8–40.2)	14.1 (11.1–17.8)
	*Vaginal delivery* a	72.6 (69.0–75.8)	79.3 (73.0–84.4)	76.1 (63.7–85.2)	76.3 (71.2–80.7)	68.6 (56.1–78.9)	75.5 (71.0–79.5)
	Women with vaginal delivery^a^	329	255	140	657	67	724
	Delivery assisted by nurse/ obstetrician	23.1 (10.6–43.3)	13.5 (5.1–31.3)	10.4 (2.0–39.4)	22.6 (13.0–36.2)	0.2 (0.0–1.3)	16.6 (8.6–29.5)
	Vertical position	96.0 (93.2–97.7)	97.0 (94.0–98.5)	89.2 (79.9–94.5)	95.2 (92.0–97.1)	93.7 (72.6–98.8)	95.0 (92.0–97.0)
	Performance of uterine fundal pressure maneuver	6.9 (5.8–8.1)	7.9 (5.8–10.7)	4.6 (1.5–13.2)	7.2 (5.7–8.9)	3.3 (1.1–9.5)	6.8 (5.4–8.5)
	Performance of episiotomy	6.4 (5.1–8.1)	8.5 (3.5–19.2)	4.1 (0.5–25.0)	6.8 (4.0–11.3)	6.1 (2.4–14.6)	6.7 (4.1–10.8)

CI: confidence interval.

a Includes instrumental delivery (use of forceps or vacuum extractor).

Compared to women living in the MR and/or small cities, the residents of the MRJ had a higher proportion of women with private source of funding, white skin color/race, aged 35 years or older, with 16 or more years of formal study, multiparous women with previous VD, earlier onset and greater PC adequacy, as well as a lower proportion of women who have traveled for delivery (Table 1). They presented a lower proportion of gestational risk, except for infections upon admission for delivery; a higher proportion of labor, VD, and intrapartum cesarean section; presence of a companion; greater use of analgesia; and delivery in vertical position and with the ON presence (Table 2).

Compared to women whose deliveries took place in institutions with public source of funding, those who used private services are in a higher proportion white, age ≥ 35 years, higher level of education, live with a partner, are nulliparous or with previous cesarean section. They start the PC early, have greater PC adequacy, and travel less for delivery (Table 1). The highest proportion of births is by cesarean section, mainly antepartum, with the presence of a companion. They refer to having been assisted by ON/OB during labor and delivery in a lower proportion. They also received, in a greater proportion, peripheral venous catheter and epidural analgesia interventions, with 69% of the women who went into labor evolving to VD (Table 2).

The variables associated with the outcome "going into labor" were: hospital delivery in the MRJ and public source of funding (distal level), being nulliparous or multiparous with previous VD (compared with multiparous women with previous cesarean section), not being obese, absence of obstetric risk, and preference for VD at the end of pregnancy (intermediate level) (Table 3).

**Table 3:** Labor associated with sociodemographic characteristics, prenatal and obstetric care, Birth in Brazil II, state of Rio de Janeiro (NBII-ERJ), 2021–2023.

		Went into labor (%)	Crude		Adjusted	
			OR	CI	OR	CI
LEVEL 1^a^		54.4				
*Sociodemographic*						
Hospital location						
	Municipality of Rio de Janeiro	63.6	2.2	(0.7–7.4)	3.7	(1.7–7.8)
	Metropolitan Region	51.1	1.3	(0.7–2.4)	1.4	(0.9–2.4)
	Other Municipalities	43.9	ref	-	ref	-
Source of funding for delivery						
	Public	66.0	7.2	(3.7–14.2)	9.7	(5.1–18.6)
	Private	21.2	ref	-	ref	-
Skin color						
	White	47.1	ref	-	ref	-
	Black	57.9	1.54	(1.1–2.2)	0.71	(0.5–1.1)
	Mixed-race	57.5	1.52	(1.1–2.1)	1.03	(0.7–1.5)
Mother's age (years)						
	10 to 19	71.8	3.3	(1.2–9.2)	1.66	(0.5–5.2)
	20 to 34	54.6	1.5	(1.0–2.3)	1.17	(0.8–1.8)
	35 or over	43.7	ref	-	ref	-
Level of education (years)						
	≤ 8 (some ES)	67.5	3.6	(2.4–5.6)	0.7	(0.3–1.6)
	9 to 11 (ES)	61.6	2.8	(1.9–4.1)	0.6	(0.3–1.2)
	12 to 15 (HS)	51.9	1.9	(1.3–2.7)	0.7	(0.4–1.2)
	≥ 16 (CD)	36.3	ref	-	ref	-
Lives with a partner						
	No	63.1	1.6	(1.23–1.96)	0.9	(0.7–1.2)
	Yes	52.4	ref	-	ref	-
LEVEL 2^b^						
*History of previous cesarean section*						
	Nulliparous	53.0	2.8	(2.1–3.6)	2.5	(1.8-3.5)
	Multiparous with previous vaginal delivery only	83.9	12.8	(8.5–19.1)	9.5	(5.3–17.4)
	Multiparous with previous cesarean section	29.1	ref	-	ref	-
*Type of delivery preference at the end of pregnancy*						
	Vaginal	71.7	5.1	(3.8–6.8)	2.7	(1.9–3.7)
	Cesarean section or without preference	33.3	ref	-	ref	-
*Prenatal care (PC)*						
PC adequacy^c^						
	No	64.4	1.8	(1.1–2.9)	1.1	(0.7–1.6)
	Yes	50.6	ref	-	ref	-
Traveling for delivery						
	No	53.0	ref	-	ref	-
	Yes	65.1	1.7	(1.05–2.6)	1.5	(0.8–2.9)
*Pregestational obesity*						
	No	58.1	2.0	(1.59–2.5)	1.5	(1.0–2.2)
	Yes	40.8	ref	-	ref	-
*Obstetric risk* d						
	No	61.1	2.4	(2.0–3.0)	3.8	(2.3-6.4)
	Yes	39.3	ref	-	ref	-

OR: odds ratio. CI: 95% confidence interval. ES: Elementary School. HS: High School. CD: College Degree.

a Level 1: adjusted by hospital location and source of funding for delivery.

b Level 2: adjusted by hospital location, source of funding for delivery, history of previous cesarean section, type of delivery preference at the end of pregnancy, pregestational obesity, and obstetric risk.

c Onset of up to 12 weeks of gestation and number of visits appropriate for gestational age at birth according to WHO criteria.

d Diabetes (chronic or gestational); gestational hypertension (chronic arterial hypertension [HA], gestational HA, preeclampsia, eclampsia, HELLP); severe chronic disease (lupus, scleroderma, kidney disease, heart disease, cancer); infections upon hospital admission; PPA; placenta accreta/increta/percreta; IUGR; twin pregnancy; HIV infection; and hospitalization during pregnancy.

The variables associated with VD were: having less than 15 years of formal study and living without a partner (distal level); being nulliparous or multiparous with previous VD only (intermediate level); presence of a doula, being able to feed and move, use of non-pharmacological methods for pain relief, being accompanied by ON/OB during labor, and use of analgesia (proximal level) (Table 4).

**Table 4:** Vaginal delivery associated with sociodemographic characteristics and those related to obstetric and prenatal care, labor and delivery, Birth in Brazil II, state of Rio de Janeiro (NBII-ERJ), 2021–2023.

		Vaginal delivery (%)	Crude		Adjusted	
			OR	CI	OR	CI
LEVEL 1^a^		75.5				
*Sociodemographic*						
Hospital location						
	Municipality of Rio de Janeiro	72.6	0.8	(0.5–1.6)	0.9	(0.5–1.9)
	Metropolitan Region	79.3	1.2	(0.6–2.5)	1.1	(0.5–2.5)
	Other Municipalities	76.1	ref	-	ref	
Source of funding for delivery						
	Public	76.3	1.5	(0.8–2.6)	0.6	(0.2–1.7)
	Private	68.6	ref	-	ref	
Skin color						
	White	75.3	ref	-	ref	
	Black	80.0	1.3	(0.8–2.3)	1.0	(0.6–1.7)
	Mixed-race	74.0	0.9	(0.6–1.5)	0.8	(0.5–1.4)
Mother's age (years)						
	10 to 19	82.0	1.4	(0.7–2.6)	1.3	(0.6–2.4)
	20 to 34	77.1	ref	-	ref	-
	35 or over	61.5	0.5	(0.3–0.9)	0.6	(0.3–1.0)
Level of education (years)						
	≤ 11 (less than ES)	75.1	2.6	(1.3–5.2)	2.6	(1.1–6.5)
	12 to 15 (HS)	82.0	4.0	(1.8–8.9)	4.6	(1.6–13.4)
	≥ 16 (CD)	53.5	ref	-	ref	-
Lives with a partner						
	No	84.5	2.0	(1.2–3.4)	1.9	(1.1–3.1)
	Yes	73.0	ref	-	ref	-
LEVEL 2^b^						
*Obstetric history*						
History of previous cesarean section						
	Nulliparous	73.1	4.6	(2.8–7.4)	2.47	(1.5–4.0)
	Multiparous with previous vaginal delivery	only 91.5	18.0	(9.4–34.2)	13.28 (5, 6–31.6)	
	Multiparous with previous cesarean section	37.4	ref	-	ref	-
*Type of delivery preference at the end of pregnancy*						
	Vaginal	80.8	2.7	(1.4–5.1)	1.8	(0.91–3.7)
	Cesarean section or without preference	61.4	ref	-	ref	-
*Prenatal care (PC)*						
PC adequacy^c^						
	No	76.6	1.1	(0.9–1.4)	0.7	(0.4–1.2)
	Yes	75.0	ref	-	ref	-
Traveling for delivery						
	No	75.1	ref	-	ref	-
	Yes	78.1	1.2	(0.4–3.4)	1.4	(0.4–5.7)
*Pregestational obesity*						
	No	77.8	1.5	(0.7–3.3)	1.2	(0.6–2.4)
	Yes	69.6	ref	-	ref	-
*Obstetric risk* d						
	No	74.1	ref	-	ref	-
	Yes	80.4	1.4	(1.0–2.1)	1.5	(0.9–2.4)
LEVEL 3^b^ *Good practices*						
Presence of a companion during labor and delivery						
	No	66.2	ref	-	ref	-
	Yes	78.7	1.9	(0.8–4.3)	2.6	(1.0–7.0)
Presence of a doula						
	No	74.8	ref	-	ref	-
	Yes	89.6	2.9	(0.9–9.2)	4.3	(1.2–15.8)
Was able to feed herself^e^						
	No	68.8	ref	-	ref	-
	Yes	88.1	3.1	(2.2–4.3)	3.1	(2.2–4.6)
Was able to move^e^						
	No	65.3	ref	-	ref	-
	Yes	85.8	3.7	(1.9–6.9)	3.46	(1.8–6.6)
Use of non-pharmacological methods^e^						
	No	69.0	ref	-	ref	-
	Yes	84.0	2.8	(1.9–4.1)	3.3	(2.12–5.1)
Labor accompanied bya nurse^e^						
	No	62.6	ref	-	ref	-
	Yes	79.7	3.9	(1.1–14.2)	3.37	(1.1–10.5)
*Interventions*						
Use of peripheral venous access^e^						
	No	74.9	ref	-	ref	-
	Yes	76.1	1.2	(0.7–2.3)	1.2	(0.5–2.9)
Use of oxytocin^e^						
	No	76.0	ref	-	ref	-
	Yes	72.6	0.8	(0.31– 2.2)	0.6	(0.2–2.1)
Use of analgesia^e^						
	No	75.1	ref	-	ref	-
	Yes	84.6	2.3	(1.0–5.0)	5.8	(1.8–18.9)
Amniotomy^e^						
	No	73.3	ref	-	ref	-
	Yes	88.6	2.2	(1.2–4.0)	1.8	(1.0–3.3)

OR: odds ratio.

CI: 95% confidence interval.

ES: Elementary School.

HS: High School.

CD: College Degree.

a Level 1: Adjusted by source of funding for delivery, age, level of education, and lives with a partner.

b Levels 2 and 3 adjusted for source of funding for delivery, age, level of education, lives with a partner, and history of previous cesarean section.

c Onset of up to 12 weeks of gestation and number of visits appropriate for gestational age at birth according to WHO criteria.

d Diabetes (chronic or gestational); gestational hypertension (chronic arterial hypertension [HA], gestational HA, preeclampsia, eclampsia, HELLP); severe chronic disease (lupus, scleroderma, kidney disease, heart disease, cancer); infections upon hospital admission; PPA; placenta accreta/increta/percreta; IUGR; twin pregnancy; HIV infection; and hospitalization during pregnancy.

e For the evaluation of good practices and interventions, we included in crude and adjusted regressions only women with labor time between 2 and 16 hours (2nd, 3rd, and 4th quintiles, as described in Table 2).

## DISCUSSION

There is inequality in labor care in the RJE by geographic area, type of funding, and sociodemographic status of the parturients. The MRJ presents better socioeconomic profile, higher proportion of labor, and greater use of some good practices recommended by the MS^8^ and the WHO^7^ — such as presence of a companion, ON/OB assistance, and vertical positions for delivery. In small cities and in the private sector, there is a higher occurrence of cesarean sections, especially antepartum ones. In small cities, we observed a higher frequency of women with obstetric risk, although the older parturients reside in the MRJ.

Good practices in labor/delivery are available in all three areas of the RJE, but the presence of a doula, companion of choice, and intake of liquids or food during labor were more frequent in women with private source of funding for delivery. In addition, we observed a higher frequency of interventions in this group of women as well as lower performance of ON/OB.

Going into labor was associated with provision of care in the MRJ, public source of funding for delivery, being nulliparous or multiparous with previous VD, having preference for VD at the end of pregnancy, and not having obesity or complications during pregnancy. Conversely, VD was associated with lower level of education and absence of a partner, being nulliparous or multiparous with previous VD, adopting good practices in labor/delivery, and analgesia, regardless of the type of funding and hospital location.

The best indicators observed in the MRJ can be understood as results of a humanization policy of labor care that began in 1994, with the inauguration of the Leila Diniz Maternity Hospital and subsequent extension of this new model of labor care to all maternity hospitals under management of the Municipal Department of Health of Rio de Janeiro. The implementation of the *Cegonha Carioca* program in 2011^5^ reduced travels and increased the comfort and safety of pregnant women. Currently, the MRJ is responsible for 90% of SUS deliveries in the city.

In the MR, the creation of large state maternity hospitals in the 2010s improved access to good practices in labor care, approaching the MRJ standard, except for the presence of a companion during labor/delivery, although this practice has significantly expanded in the RJE. The law that guarantees this right dates from 2005^11^, but in Brazil, in 2011, only 18% of the parturients had the full-time presence of a companion of their choice^12^. In this study, this value reaches 80% in the MRJ/small cities and 98% in the private sector.

ON/OB assisted more than 60% of women's labor in the RJE, except for the private sector. However, ON/OB delivery assistance was only 16.6%, being more frequent in the MRJ and public services. This indicates that there is room to increase the presence of these professionals in RJE maternity hospitals. Since 1998, the MRJ has cooperated with the School of Nursing of Universidade Estadual do Rio de Janeiro (UERJ) to qualify ON for prenatal and delivery care, and these professionals were absorbed by RJE maternity hospitals^13^. The importance of ON/OB in labor was highlighted by several studies, including an article published in the *Lancet* journal, whose authors highlighted their role in optimizing the physiology of delivery, reducing interventions and improving outcomes^14^. Nove et al.^15^ estimated that more than 60% of maternal, neonatal, and fetal deaths in low-income countries could be avoided with the presence of these professionals^16^.

The use of oxytocin to accelerate labor and amniotomy halved compared to national data of the NB research, the first Brazilian perinatal survey conducted in 2011, while Uterine fundal pressure maneuver and episiotomy had an 80% reduction^9^. Lithotomy, reported by 92% of Brazilians in the aforementioned research^9^, was replaced in the RJE with the vertical position to deliver (95%), without distinction by area or type of funding. The vertical position (sitting, leaned back, squatting, or standing) is recommended to shorten the expelling period and reduce episiotomy and the use of forceps^17,18^.

All these changes were also influenced by the *Rede Cegonha* program, developed by the Federal Government as of 2011, with the key objective of humanizing labor care in the maternity hospitals of the SUS^19,20^. Nonetheless, we should mention the organized movement of women, especially in the informed middle class. Their struggle for respect for physiology, the right to choose the type of delivery and autonomy in the conduct of the birth of their child influenced private services to change conduct and adopt practices based on scientific evidence^21,22^.

In the private sector, it is observed that when the parturient goes into labor, it evolves to VD almost as much as in the public sector. Nevertheless, the model of the antepartum cesarean section is widespread and a difficult practice to change, even with initiatives — such as *Parto Adequado* [Adequate Delivery]^23^, an initiative of the Brazilian Federal Government aiming at the development and implementation of good practices and improvement of labor care —, which have been dedicated to the valorization of vaginal delivery and to the reduction of the percentage of unnecessary cesarean sections in supplementary health. One of the consequences of antepartum cesarean sections is the excess of births with 37–38 gestational weeks, called early term. According to the NB research, almost half of the private-sector babies were born early, and adverse outcomes, such as transient tachypnea, hospitalization in Neonatal Intensive Care Unit, jaundice, and infections, were more frequent^24^.

The presence of a doula during labor/delivery, more frequent in the MRJ and the private sector, provides continuous support, encouragement, facilitates the communication of women with the health team, and contributed, in this study, to the occurrence of VD^25^. The presence of women's companion of choice, although it lost significance after adjustment for other variables, is also an important measure to increase social support in labor as well as to resume birth as a family event^12^.

Being seen in public service and having a previous VD were the main variables that contributed to the "going into labor" outcome in the RJE. Women's preference for VD at the end of pregnancy was also an important factor for them to going into labor. Authors of studies on the topic show the relevance of supporting and promoting VD during PC^26^. It should be noted that women's preference was not associated with VD, evidencing that other issues, such as the model of care with the use of good practices, are more relevant for the "delivery" outcome.

In this study, the use of good practices in labor increased the chance of women having a VD, a result consistent with the literature on the topic. Most studies on the effects of good obstetric practices conducted in developed countries also apply to the Brazilian reality^7^. VD was associated with obstetric history (nulliparity or history of vaginal delivery), being a relevant result, considering that one of the main strategies for reducing cesarean sections in Brazil is the prevention of the first cesarean section, avoiding repeating cesarean sections. Finally, analgesia in labor was also associated with VD, being a useful technology for relieving delivery pain, and may be an ally in reducing cesarean sections^27^. The offer of epidural analgesia brings comfort to the parturient and should be expanded, especially in the SUS, where it is little offered. Fear of pain is the main reason for the desire for cesarean section^26^, and advances in techniques and medicines have made analgesia safer for the mother and the baby^27,28^.

The strengths of this research are the study design, which enabled the representation of all large geographic areas of the RJE, and type of funding for delivery; the representative number of women; and the low rate of refusal (data not shown), contributing to the robustness of the study. As limitations, the results do not apply to hospitals with less than one hundred deliveries/year; the public source of funding includes deliveries in public and private hospitals linked to SUS, and the analysis did not distinguish between these two types of services; and variables related to complications are derived from medical records, depending on the quality of the record in each hospital. The absence of registration was considered absence of complication. Studies whose authors validate the absence of registration in the medical records as absence of complications and that improve the measurement of the onset and duration of PC are essential for future research.

We conclude that there have been advances in labor care in the RJE, especially the MRJ, although the frequency of labor and VD remains low. The adoption of good practices was positively associated with having VD, with greater magnitude in the presence of doulas and the use of analgesia. Women in social vulnerability conditions had a higher chance of VD. Our results point to the main practices promoting VD in the RJE and can subsidize the improvement of care.

## Data Availability

The datasets generated and/or analyzed during the study are available from the corresponding author upon request.
