# Supplementary material for: Inequalities in the care for Labor and Delivery in Rio de Janeiro – Birth in Brazil Research II: national survey on abortion, delivery, and birth
Source: Rev Saude Publica. 2025 Oct 20;59(Suppl 1):e4s. doi: 10.11606/s1518-8787.2025059006516 (PMC12539634; doi:10.11606/s1518-8787.2025059006516)
Supplement: Translation

# Translation: Desigualdades na atenção ao Trabalho de Parto e Parto no Rio de Janeiro – Nascer no Brasil II: pesquisa nacional sobre aborto, parto e nascimento

**Authors:** Maria do Carmo Leal, Ana Paula Esteves-Pereira, Rosa Maria Soares Madeira Domingues, Sonia Duarte de Azevedo Bittencourt, Mariza Miranda Theme-Filha, Neide Pires Leal, Marcos Nakamura-Pereira, Marcos Augusto Bastos Dias, Thaiza Dutra Gomes de Carvalho, Tatiana Henriques Leite, Silvana Granado Nogueira da Gama

**Affiliations:** I Fundação Oswaldo Cruz. Escola Nacional de Saúde Pública. Departamento de Métodos Quantitativos em Saúde. Rio de Janeiro, RJ, Brasil; II Fundação Oswaldo Cruz. Instituto Nacional de Infectologia Evandro Chagas. Laboratório de Pesquisa Clínica em DST/Aids. Rio de Janeiro, RJ, Brasil; III Fundação Oswaldo Cruz. Instituto Nacional da Saúde da Mulher da Criança e do Adolescente Fernandes Figueira. Rio de Janeiro, RJ, Brasil; IV Universidade do Estado do Rio de Janeiro. Instituto de Medicina Social. Departamento de Epidemiologia. Rio de Janeiro, RJ, Brasil

**Keywords:** Parto, Trabalho de Parto, Setor Público, Setor Privado, Brasil

## Abstract

**OBJETIVOS:**

Descrever a atenção ao trabalho de parto (TP) e parto no estado do Rio de Janeiro (ERJ) segundo a localização do hospital e o tipo de financiamento do parto e verificar os fatores sociais, geográficos e assistenciais associados a entrar em trabalho de parto (TP) e a ter parto vaginal (PV).

**MÉTODOS:**

Estudo seccional de base hospitalar ("Nascer no Brasil II: Pesquisa nacional sobre aborto, parto e nascimento") realizado em 29 hospitais do ERJ. Foram elegíveis puérperas com nascidos vivos e/ou natimortos com idade gestacional ≥ 22 semanas ou peso ≥ 500 g, no total de 1.762 mulheres. As entrevistas foram realizadas nos hospitais, no puerpério imediato, e foram extraídos dados do cartão de pré-natal e do prontuário materno. Foi realizada regressão logística múltipla para os desfechos TP e PV, sendo utilizado um modelo hierarquizado, com estimativa das razões de chance e respectivos intervalos de confiança.

**RESULTADOS:**

A frequência de TP foi de 54% e de PV 41%. Mostraram associação com entrar em TP ser atendida em hospitais do município do Rio de Janeiro (MRJ), com financiamento público, ser nulípara ou multípara com PV anterior, ter preferência pelo PV no fim da gestação, não ser obesa nem ter apresentado intercorrências na gestação. Ter PV foi associado com menos anos de estudo, ausência de companheiro, ser nulípara ou multípara com PV anterior, ter acesso às boas práticas no TP e parto e ao uso de analgesia no TP, independentemente do tipo de financiamento e da localização do hospital.

**CONCLUSÃO:**

Foram verificados avanços na assistência ao parto no ERJ, embora a frequência de TP e de PV ainda seja baixa, bem como a de boas práticas, com melhores resultados para o MRJ. Todas as boas práticas se associaram ao PV, destacadamente o uso de analgesia e a presença de doulas. O PV foi mais frequente em mulheres socialmente vulneráveis.

## INTRODUÇÃO

O estado do Rio de Janeiro (ERJ) apresenta a terceira maior população do país, 16.055.164 habitantes, segundo o censo demográfico de 2022. Concentra, em sua região metropolitana (RM), 75% da população do estado, da qual mais da metade, no município do Rio de Janeiro (MRJ)^1^.

A história da constituição do ERJ foi forte determinante das características da saúde pública no estado. Com a herança do período em que foi capital do Brasil, o MRJ conta com uma estrutura de serviços públicos de saúde muito distinta de todos os demais municípios.

Desde o final dos anos 1990, o MRJ desenvolve uma política de humanização da assistência ao parto, com promoção das boas práticas na atenção ao TP/P^2^. Essas medidas incluem reforma do espaço físico das maternidades, visando a um ambiente mais confortável e acolhedor; protocolos assistenciais para a adoção de boas práticas e abandono de práticas prejudiciais; incorporação da enfermagem obstétrica (EO) na atenção ao parto; e estímulo à presença do acompanhante, antes mesmo da publicação da lei federal^3,4^. Posteriormente, em 2011, com a implantação do programa "Cegonha Carioca", foi definida toda a rede de referência das maternidades para atenção ao parto de gestantes com acompanhamento pré-natal (PN) em unidades públicas, com garantia de vaga e de transporte da casa para o hospital no momento do parto^5,6^.

Há 12 anos, o estudo "Nascer no Brasil" (NB) já identificava que a atenção obstétrica no país era intervencionista, sem respeito à fisiologia do parto nem prioridade para a autonomia da mulher, na contramão das recomendações da Organização Mundial da Saúde (OMS)^7^ e do Ministério da Saúde (MS)^8^, que tomavam como base as evidências científicas já disponíveis àquela época de promoção das boas práticas e abandono de intervenções desnecessárias^9^.

A hipótese deste artigo é que o MRJ apresenta maior frequência de boas práticas na assistência ao trabalho de parto (TP) e parto em comparação às demais regiões do ERJ.

Por esse motivo, este estudo tem por objetivos:

- descrever a atenção ao parto no ERJ segundo a localização do hospital (MRJ; demais municípios da RM); e municípios do interior do Estado) e o financiamento do parto;
- verificar as desigualdades sociais, geográficos e assistenciais e sua associação com entrar em TP e ter parto vaginal (PV).

## MÉTODOS

### Pesquisa Nascer no Brasil II

Estudo transversal de base hospitalar, abrangência nacional e com duas ondas de seguimento ("Nascer no Brasil II: pesquisa nacional sobre aborto, parto e nascimento)", no período 2021–2024. O protocolo do estudo NBII encontra-se publicado em Leal et al.^10^.

### Estudo no Estado do Rio de Janeiro

#### Delineamento e Local

Estudo transversal de base hospitalar em 29 hospitais com ≥ 100 nascidos vivos [NV]/ano (segundo o Sistema de Informação sobre Nascidos Vivos [SINASC]-2017), localizados em 18 municípios do ERJ.

#### População e Período

Mulheres admitidas para parto e seus recém-nascidos (RN) entre novembro de 2021 e junho de 2023.

#### Cálculo Amostral

A amostra foi calculada com base na proporção de cesarianas no ERJ em 2019 (57%), nível de significância de 5% e potência de 90%, para detectar diferenças de 7%. Utilizou-se um efeito de desenho de 1,3, resultando no tamanho amostral mínimo de 1.350 mulheres admitidas para parto. Cálculos *post hoc* demonstram que a amostra tem poder de detectar diferenças absolutas de 5% para desfechos com prevalência entre 5 e 20%.

#### Desenho Amostral

Foi selecionada uma amostra probabilística em dois estágios:

- Estágio 1: Os hospitais foram estratificados pela localização (MRJ, RM e interior), tipo (público, misto, privado) e tamanho (100–499, ≥ 500 NV/ano). Do total de 120 hospitais com ≥ 100 NV/ano no ERJ (segundo o SINASC-2017), 29 foram sorteados para a pesquisa;
- Estágio 2: A identificação das mulheres ocorreu por meio dos registros hospitalares (censo e livro de internações) que, em seguida, foram registradas em uma lista única, organizada em ordem cronológica pela data do parto. A seleção das participantes foi realizada de forma consecutiva até atingir o número amostral estipulado: 30 mulheres nos hospitais com 100–499, 50 mulheres nos hospitais privados com ≥ 500 NV/ano e 90 nos públicos e mistos com ≥ 500 NV/ano.

#### Critérios de Seleção

Foram incluídas puérperas com parto de NV com qualquer peso ou idade gestacional, e de natimortos com idade gestacional ≥ 22 semanas ou peso ≥ 500 g. Foram excluídas mulheres com parto trigemelar ou mais, as com dificuldade de comunicação (estrangeiras, indígenas que não entendiam português, pessoa com deficiência auditiva ou com doença mental severa), e as internadas para parto por determinação judicial. No total, foram entrevistadas 1.762 mulheres admitidas para parto, sendo 1.752 NV e dez natimortos.

Foram realizadas entrevistas face a face no puerpério imediato, e foram fotografados os cartões de PN, quando disponíveis, e extraídos os dados clínicos do prontuário hospitalar.

#### Variáveis de Desfecho

Analisaram-se duas variáveis desfecho:

- entrar em TP;
- ter PV.

Foram classificadas como tendo entrado em TP todas as mulheres com TP de início espontâneo ou induzido que alcançaram dilatação do colo de útero ≥ 4 cm.

Para o desfecho PV, foram consideradas todas as mulheres com PV, incluindo aquelas com uso de fórceps ou vácuo extrator.

#### Variáveis de Exposição para o Desfecho "Entrar em TP"

Localização geográfica do hospital (MRJ, RM, interior); fonte de pagamento do parto (pública, privada); cor da pele/raça autorrelatada (branca, preta, parda, amarela, indígena, categorias utilizadas pelo Instituto Brasileiro de Geografia e Estatística); idade (< 20, 20–34, ≥ 35 anos); anos de estudo (≤ 7, 8–10, 11–14, ≥ 15); vive com companheiro (não, sim); história obstétrica (nulípara, multípara apenas com vaginal, multípara com cesariana); adequação do PN (não, sim); preferência pelo tipo de parto ao fim da gestação (vaginal, cesariana, sem preferência); buscou mais de uma maternidade para o parto — peregrinação (não, sim); obesidade (índice de massa corporal — IMC < 30, IMC ≥ 30) e risco obstétrico (não, sim); tempo de admissão para o parto.

Mulheres com parto em hospitais públicos ou privados conveniados ao Sistema Único de Saúde (SUS) sem pagamento da internação por plano de saúde foram classificadas como tendo "fonte de pagamento pública"; as com parto em hospitais privados e pagamento por plano de saúde ou desembolso direto, como tendo "fonte de pagamento privada".

Para a classificação da adequação do PN, utilizou-se o início até 12 semanas de gestação e o número de consultas segundo a IG, conforme o seguinte calendário: IG ≥ 20 e < 26 = consultas ≥ 2; IG ≥ 26 e < 30 = consultas ≥ 3; IG ≥ 30 e < 34 = consultas ≥ 4; IG ≥ 34 e < 36 = consultas; ≥ 5; IG ≥ 36 e < 38 = consultas ≥ 6; IG ≥ 38 e < 40 = consultas ≥ 7; IG ≤ 40 = consultas ≥ 8^3^.

Definiu-se o critério de risco obstétrico como a presença de algumas das seguintes condições: gestação gemelar, hipertensão arterial (HA) crônica; HA gestacional; pré-eclâmpsia; eclâmpsia; síndrome HELLP (pré-eclâmpsia grave com hemólise, enzimas hepáticas elevadas, baixa contagem de plaquetas), diabetes pré-gestacional; diabetes gestacional; doença crônicas grave (lúpus, esclerodermia, doença renal, doença cardíaca, câncer); infecções no diagnóstico da internação; descolamento prematuro de placenta (DPP); placenta acreta/increta/percreta e crescimento intrauterino restrito (CIUR); e internação por qualquer motivo durante a presente gestação.

Para o cálculo do tempo entre a admissão hospitalar e o parto, considerou-se o intervalo entre a data e hora da internação hospitalar e a data e a hora do parto, com posterior categorização em quintis: quintil 1 (< 2 h), quintil 2 (2 h a < 5 h), quintil 3 (5 h a < 8 h), quintil 4 (8 h a < 16), quintil 5 (≥ 16 h). Excluímos das análises das regressões brutas e ajustadas as mulheres do primeiro e do último quintil. As primeiras não tiveram tempo suficiente para receberem as boas práticas e as últimas apresentaram um período prolongado, sugerindo a presença de complicações que poderiam interferir na adesão às boas práticas.

#### Variáveis de Exposição para o Desfecho "Parto Vaginal"

Todas as covariáveis descritas anteriormente foram utilizadas e incluiu-se variáveis relacionadas às boas práticas do TP: presença de acompanhante durante o TP/parto, presença de doula, ingestão de líquidos ou alimentos durante o TP, liberdade de movimentação durante o TP, uso de métodos não farmacológicos para alívio da dor e atuação da enfermeira obstétrica/obstetriz (EO/O) no TP/parto. Considerou-se como intervenções durante o TP: uso de cateter venoso periférico, uso de ocitocina (hormônio utilizado para a indução e/ou aceleração do TP), amniotomia (ruptura artificial das membranas ovulares), uso de analgesia no TP (peridural ou raquidiana), litotomia (posição dorsal no parto) e manobra de Kristeller (pressão externa sobre o útero da mulher).

As informações utilizadas para a classificação do risco obstétrico, trabalho de parto, tipo de parto, tempo entre admissão e parto, uso de ocitocina, amniotomia e analgesia no TP foram obtidas no prontuário hospitalar. As demais variáveis foram obtidas durante a entrevista com a puérpera.

#### Análise dos Dados

As análises descritivas dos dados foram estratificadas pela localização dos hospitais onde as parturientes foram atendidas e pela fonte de financiamento do parto. As proporções e seus intervalos de confiança de 95% (IC95%) foram estimadas.

Um modelo teórico foi utilizado para classificar as variáveis explanatórias de acordo com o seu nível hierárquico em relação ao desfecho (distal, intermediário e proximal).

- Variáveis distais: idade, cor da pele, anos de estudo, vive com companheiro, fonte de financiamento do parto;
- Intermediárias: história obstétrica, adequação do PN, peregrinação para o parto, obesidade, risco obstétrico, preferência pelo tipo de parto no final da gestação;
- Proximais: presença de acompanhante, de doula, de EO/O, alimentação, liberdade de movimentação, uso de métodos não farmacológicos para alívio da dor, uso de acesso venoso periférico, ocitocina, analgesia peridural, amniotomia, litotomia e manobra de Kristeller.

Para a análise do desfecho "entrar em TP", entraram no modelo somente as variáveis distais e intermediárias. Para a análise do desfecho "PV", foram incluídas variáveis dos três níveis, mas apenas mulheres que apresentaram TP assistido no hospital foram incluídas na análise, sendo excluídas as que tiveram indicação de cesariana no momento da admissão hospitalar.

Em cada nível, as variáveis foram selecionadas pelo critério de p < 0,20 para sua permanência no modelo. Para as análises seguintes foram mantidas no modelo aquelas que apresentaram associação (p < 0,05) com os desfechos, após ajuste pelas variáveis de mesmo nível e de níveis hierárquicos mais elevados. Foi calculada a razão de chances bruta e ajustada e IC95%.

Todas as análises foram realizadas com o *software* IBM SPSS Statistics for Windows, Versão 23.0 (IBM Corp. Armonk, NY, USA), com inclusão do efeito de desenho, ponderação e calibração da amostra. Para a calibração, foram utilizados grupos formados pela combinação de estrato, tipo de parto (vaginal ou cesariana) e idade da mulher (10–19, 20–34, ≥ 35), tendo como referência o SINASC-2022.

A pesquisa NBII foi aprovada pela Comissão Nacional de Ética em Pesquisa (CONEP), com Certificado de Apresentação para Apreciação Ética (CAAE): 21633519.5.0000.5240, em 11 de março de 2020, e teve a aprovação dos comitês locais de ética em pesquisa das instituições ou da diretoria clínica quando na ausência destes.

## RESULTADOS

No ERJ, os partos estão concentrados no MRJ e demais municípios da RM, com um quarto ocorrendo em municípios do interior, tendo 74% financiamento público. A maioria das mulheres é de cor/raça parda; tem de 20 a 34 anos; apresenta 12 ou mais anos de estudo; e vive com companheiro. A maioria tem partos anteriores, inicia o PN até 12 semanas gestacionais e tem PN adequado, com 12% referindo peregrinação (Tabela 1). Um terço das mulheres apresentou algum risco obstétrico e um quarto apresentou obesidade. Aproximadamente 50% entraram em TP, com 41% tendo PV e 71% a presença de acompanhante. A maioria dos partos ocorreu até as primeiras 8 horas de internação. Em mulheres com TP, mais de 60% tiveram acompanhante e foram assistidas por enfermeiras, mas menos de 50% se alimentaram, tiveram liberdade de movimentação e usaram métodos não farmacológicos. A intervenção mais frequente foi o uso de acesso venoso (48%), com menos de 15% das mulheres tendo uso de ocitocina, analgesia e amniotomia. Quase a totalidade das mulheres teve parto em posições verticalizadas, 17% com a assistência por EO e 7% foram submetidas à manobra de Kristeller e episiotomia (Tabela 2).

**Tabela 1:** Características sociodemográficas, da história obstétrica e da atenção pré-natal, segundo localização do hospital e financiamento do parto, Pesquisa Nascer no Brasil II, estado do Rio de Janeiro (NBII-ERJ), 2021–2023.

		Município do Rio de Janeiro	Região Metropolitana	Outros Municípios	Público	Privado	TOTAL
		% (IC)	% (IC)	% (IC)	% (IC)	% (IC)	% (IC)
Total de mulheres pós-parto		711	631	420	1.305	457	1.762
*Sociodemográficas*							
Localização do Hospital							
	Município do Rio de Janeiro	-	-	-	36,2 (8,3–77,9)	52,1 (26,1–76,9)	40,4 (15,6–71,1)
	Região Metropolitana	-	-	-	36,0 (12,5–68,9)	34,9 (14,8–62,2)	35,8 (16,1–61,7)
	Outros Municípios	-	-	-	27,6 (12,8–49,8)	12,9 (6,7–23,5)	23,8 (12,3–41,1)
Financiamento do Parto							
	Público	66,5 (25,0–92,1)	74,6 (57,1–86,7)	85,9 (78,7–90,9)	-	-	74,0 (61,6–83,5)
	Privado	33,4 (7,8–74,9)	25,3 (13,2–42,8)	14,1 (9,7–21,2)	-	-	25,9 (16,4–38,3)
Cor da pele							
	Branca	33,1 (23,4–44,5)	24,6 (19,3–30,8)	34,4 (21,7–49,8)	23,3 (19,0–28,3)	50,5 (43,7–74,3)	30,4 (26,0–35,1)
	Preta	21,5 (13,5–32,5)	23.0 (19,6–26,7)	15,8 (11,1–22,2)	24,7 (21,2–28,5)	9,3 (6,4–13,1)	20,7 (16,9–25,1)
	Parda	45,4 (40,1–50,7)	52,4 (48,3–56,6)	49,8 (39,8–59,7)	52.0 (46,8–57,2)	40,2 (32,8–48,1)	49,0 (45,2–52,7)
Idade da mãe (anos)							
	10 a 19	10,6 (6,1–18,0)	9,7 (7,0–13,4)	10,4 (7,0–15,3)	13,4 (11,2–15,9)	1,4 (0,5–3,8)	10,3 (7,9–13,2)
	20 a 34	67,4 (63,5–71,0)	74,8 (70,5–78,6)	73,3 (68,6–77,6)	73,4 (70,3–76,3)	65,7 (61,2–69,9)	71,4 (69,2–73,6)
	35 ou mais	21,9 (14,1–32,2)	15,3 (11,5–20,2)	16,1 (13,8–18,7)	13,1 (11,7–14,6)	32,7 (28,4–37,3)	18,2 (15,9–20,7)
Escolaridade (anos)							
	≤ 8 (EF incompleto)	12,1 (7,0–20,2)	15,2 (11,0–20,5)	17,1 (11,5–24,7)	19,1 (15,9–22,8)	0,9 (0,5–1,7)	14,4 (11,9–17,3)
	9 a 11 (EF completo)	30,7 (15,6–51,4)	25,2 (20,7–30,4)	29,5 (25,9–33,3)	35,5 (28,2–43,5)	8,4 (5,1–13,6)	28,5 (20,9–37,4)
	12 a 15 (EM completo)	33,3 (26,9–40,4)	46,9 (42,0–51,8)	44,4 (38,3–50,7)	40,1 (33,2–47,5)	42,8 (34,9–51,1)	40,8 (35,2–46,7)
	≥ 16 (ES completo)	23,6 (9,3–48,3)	12,5 (6,0–24,2)	8,8 (5,4–13,9)	5,1 (3,5–7,2)	47,7 (36,9–58,7)	16,1 (11,5–22,0)
	Vive com companheiro	80,8 (69,9–88,4)	79,4 (71,6–85,4)	83,9 (78,9–87,9)	76,5 (72,6–80,1)	93,9 (89,7–96,5)	81,0 (76,6–84,8)
*História obstétrica*							
Paridade							
	Nulípara	46,8 (39,7–53,9)	42,0 (35,3–49,1)	40,8 (33,2–48,8)	39,4 (35,9–43,0)	55,8 (49,1–62,3)	43,6 (40,2–47,2)
	1 a 2 partos anteriores	43,7 (40,6–46,7)	46,8 (42,0–51,3)	47,5 (41,9–53,2)	47,4 (44,8–50,1)	40,5 (35,1–46,3)	45,6 (43,3–48,0)
	3 ou mais partos anteriores	9,5 (5,8–15,2)	11,2 (8,7–14,3)	11,5 (9,3–14,2)	13,1 (11,8–14,5)	3,5 (2,0–6,1)	10,6 (9,0–12,4)
História de cesariana anterior							
	Primípara	46,8 (39,7–53,9)	42,0 (35,3–49,1)	40,8 (33,2–48,8)	39,4 (35,9–43,0)	55,8 (49,1–62,3)	43,6 (40,2–47,2)
	Multípara com cesariana anterior	24,1 (19,5–29,1)	28,7 (23,9–33,8)	38,7 (33,1–44,5)	27,7 (23,2–32,8)	33,1 (28,5–38,0)	29,1 (25,2–33,4)
	Multípara apenas com vaginal anterior	29,1 (19,2–41,5)	29,3 (22,6–37,1)	20,5 (14,1–28,8)	32,8 (28,6–37,2)	11,1 (7,5–16,2)	27,1 (22,3–32,6)
*Pref. tipo parto final gestação*							
	Vaginal	56,7 (44,2–68,5)	55,4 (46,2–64,3)	51,6 (44,8–58,4)	63,8 (59,9–67,5)	30,1 (23,9–37,1)	55,0 (49,9–60,1)
	Cesariana ou sem preferência	43,3 (31,5–55,8)	44,6 (35,7–53,8)	48,4 (41,6–55,2)	36,2 (32,5–40,1)	69,9 (62,9–76,1)	45,0 (39,9–50,1)
*Atenção pré-natal*							
Trimestre de Início do Pré-Natal							
	Primeiro Trimestre (até 12 sem)	84,0 (75,7–89,9)	75,6 (70,6–79,9)	79,6 (68,9–87,3)	74,7 (70,6–78,3)	94,9 (92,1–96,7)	80,0 (76,9–82,7)
	Segundo Trimestre (13 a 27 sem)	15,8 (9,9–24,2)	21,9 (18,1–26,3)	17,3 (11,5–25,2)	23,2 (20,7–25,8)	4,7 (3,1–7,1)	18,3 (16,0–20,9)
	Terceiro Trimestre (≥ 28 sem)	0,1 (0,0–1,1)	2,3 (1,5–3,6)	2,9 (1,2–6,8)	2,0 (0,9–4,3)	0,3 (0,0–2,5)	1,6 (0,7–3,2)
	Adequação do pré-natal^a^	72,9 (58,8–83,5)	60,9 (54,9–66,6)	67,7 (58,9–75,4)	60,1 (56,4–63,6)	89,7 (84,8–93,1)	67,4 (63,8–70,8)
	Peregrinação para o parto	5,6 (2,9–10,5)	17,9 (12,6–24,8)	13,8 (4,6–34,7)	15,5 (9,4–24,5)	1,9 (0,9–4,0)	12,0 (7,8–17,8)

IC: intervalo de confiança.

EF: ensino fundamental.

EM: ensino médio.

ES: ensino superior.

a Início até 12 semanas de gestação e número de consultas adequado para a idade gestacional no nascimento segundo critério da OMS.

**Tabela 2:** Características de risco obstétrico, tipo de parto, boas práticas e intervenções durante o trabalho de parto (TP) e parto, segundo localização do hospital e financiamento do parto, Pesquisa Nascer no Brasil II, estado do Rio de Janeiro (NBII-ERJ), 2021–2023.

		Município do Rio de Janeiro	Região Metropolitana	Outros Municípios	Público	Privado	TOTAL
		% (IC)	% (IC)	% (IC)	% (IC)	% (IC)	% (IC)
Total de mulheres pós-parto		711	631	420	1.305	457	1.762
*Obesidade pré- gestacional*		22,1 (18,3–26,4)	25,5 (18,7–33,6)	27,2 (19,9–36,1)	24,6 (19,5–30,6)	24,2 (20,0–29,0)	24,5 (20,5–28,9)
*Risco obstétrico*							
	Diabetes ou síndromes hipertensivas	17,5 (15,6–19,7)	25,6 (12,6–44,9)	27,8 (19,6–37,7)	24,2 (15,6–35,7)	19,0 (15,0–23,7)	22,9 (16,4–31,0)
	Doença crônica grave	0,6 (0,3–1,4)	0,6 (0,3–1,3)	0,8 (0,2–3,2)	0,7 (0,3–1,3)	0,7 (0,2–2,3)	0,7 (0,4–1,2)
	Infecções no diagnóstico da internação	5,3 (2,6–10,6)	1,9 (0,8–4,5)	3,6 (1,4–9,1)	4,7 (2,6–8,5)	0,7 (0,2–2,8)	3,7 (1,9–7,1)
	Descolamento prematuro de placenta	0,9 (0,5–1,5)	1,5 (0,5–4,5)	0,8 (0,4–1,6)	1,3 (0,7–2,5)	0,5 (0,1–1,7)	1,1 (0,6–2,0)
	Placenta acreta/ increta/percreta	0,4 (0,1–1,8)	0,5 (0,1–1,4)	0,5 (0,1–2,4)	0,4 (0,1–1,2)	0,5 (0,2–1,6)	0,5 (0,2–1,0)
	Crescimento intrauterino restrito	0,9 (0,2–3,5)	0,7 (0,3–1,8)	2,3 (1,6–3,3)	1,2 (0,6–2,3)	1,2 (0,6–2,6)	1,2 (0,7–2,0)
	Gestação gemelar	1,0 (0,2–4,0)	1,9 (1,0–3,6)	1,1 (0,4–2,8)	1,1 (0,4–2,9)	1,9 (0,8–4,1)	1,3 (0,6–2,7)
	Diagnóstico de HIV	0,2 (0,0–1,4)	1,5 (0,6–4,0)	0,6 (0,2–2,0)	1,0 (0,3–2,8)	0,3 (0,0–1,3)	0,8 (0,3–2,0)
	Internação durante a gravidez	2,0 (1,4–2,7)	2,2 (0,9–5,1)	4,7 (1,7–12,2)	3,1 (1,7–5,7)	1,5 (0,8–2,7)	2,7 (1,6–4,5)
	Quaisquer riscos acima	26,8 (24,1–29,6)	31,5 (15,4–53,8)	36,6 (28,6–45,3)	33,2 (23,4–44,6)	24,0 (20,1–28,4)	30,8 (23,5–39,2)
*Trabalho de parto*							
	Entrou em trabalho de parto	63,5 (36,1–84,3)	51,0 (39,9–62,1)	43,9 (34,5–53,6)	66,0 (53,3–76,7)	21,2 (15,2–28,7)	54,4 (41,2–66,9)
*Tipo de parto*							
	Parto vaginal^a^	46,1 (28,7–64,5)	40,4 (30,6–51,1)	33,4 (23,9–44,4)	50,3 (43,3–57,4)	14,5 (10,3–20,0)	41,0 (32,6–50,0)
	Cesariana	53,8 (35,4–71,2)	59,5 (48,8–69,3)	66,5 (55,5–76,0)	49,6 (42,5–56,6)	85,4 (79,9–89,6)	58,9 (49,9–67,3)
	Cesariana anteparto	36,4 (15,6–63,8)	48,9 (37,8–60,0)	56,0 (46,3–65,4)	33,9 (23,2–46,6)	78,7 (71,2–84,7)	45,5 (33,0–58,7)
	Cesariana intraparto	17,4 (10,9–26,6)	10,5 (7,9–13,9)	10,5 (6,9–15,5)	15,6 (10,9–22,0)	6,6 (3,8–11,1)	13,3 (9,1–19,0)
	Mulheres com TP	452	322	185	862	97	959
*Tempo entre internação e parto (vaginal ou cesariana intraparto)*							
	Quintil 1 (< 2 h)	18,9 (16,6–21,4)	20,8 (15,9–26,9)	21,4 (11,1–37,2)	20,1 (16,8–24,0)	18,8 (11,1–30,1)	20,0 (16,9–23,6)
	Quintil 2 (2 h a < 5 h)	19,8 (16,8–23,2)	22,2 (17,3–27,9)	24,5 (19,9–29,7)	20,7 (18,1–23,5)	28,9 (18,9–41,4)	21,5 (18,7–24,6)
	Quintil 3 (5 h a < 8 h)	20,9 (18,0–24,0)	14,9 (12,5–17,8)	15,1 (10,6–21,0)	17,4 (13,6–21,9)	21,2 (13,0–32,6)	17,8 (14,4–21,7)
	Quintil 4 (8 h a < 16)	20,7 (18,1–23,6)	19,6 (16,6–23,1)	18,7 (15,6–22,2)	19,7 (18,3–21,3)	22,2 (15,0–31,6)	20,0 (18,4–21,6)
	Quintil 5 (≥ 16 h)	19,7 (17,4–22,3)	22,4 (16,6–29,6)	20,4 (13,2–30,0)	22,1 (19,1–25,3)	8,9 (3,6–20,6)	20,8 (18,1–23,7)
*Boas práticas*							
	Acompanhante no	88,8	50,2	82,4	71,9	98,8	74,6
	TP e parto	(83,1–92,8)	(30,5–69,9)	(62,9–92,8)	(53,0–85,3)	(93,2–99,8)	(58,7–85,9)
	Presença de doula	4,3 (3,1–5,9)	0,8 (0,1–4,3)	3,8 (1,5–9,7)	2,3 (1,1–4,5)	4,7 (2,8–8,0)	2,9 (1,9–4,5)
	Pôde se alimentar	34,9 (28,5–41,8)	34,0 (22,6–47,7)	34,9 (28,7–41,7)	33,7 (28,4–39,5)	42,6 (33,1–52,6)	34,6 (29,5–40,1)
	Movimentação	53,8 (45,8–61,6)	46,7 (38,8–54,9)	44,1 (28,4–61,0)	43,3 (39,4–47,2)	44,5 (29,3–60,8)	43,4 (39,4–47,4)
	Uso de métodos não farmacológicos	43,1 (39,5–46,8)	42,5 (36,1–49,1)	45,4 (32,4–59,1)	49,9 (44,5–55,3)	46,7 (33,1–60,8)	49,5 (44,4–54,7)
	TP acompanhado por enfermeira	61,1 (53,3–68,4)	61,3 (49,5–71,9)	66,0 (50,4–78,7)	65,9 (59,8–71,5)	38,0 (24,7–53,5)	62,3 (56,4–67,9)
*Intervenções*							
	Uso de acesso venoso periférico	47,9 (41,2–54,7)	45,0 (38,6–51,5)	51,9 (45,7–58,0)	45,1 (42,3–48,0)	70,4 (61,5–78,0)	47,7 (44,2–51,3)
	Uso de ocitocina	11,9 (6,5–20,6)	14,5 (11,1–18,7)	17,0 (7,8–33,3)	12,5 (8,5–18,1)	24,4 (14,2–38,7)	13,7 (9,5–19,5)
	Uso de analgesia	5,3 (1,1–22,2)	3,5 (1,6–7,2)	1,9 (0,7–4,7)	1,2 (0,4–3,0)	29,5 (17,2–45,8)	4,0 (1,9–8,4)
	Amniotomia	15,1 (10,0–22,2)	16,5 (11,6–23,1)	7,4 (5,1–10,7)	12,8 (10,3–15,8)	25,5 (14,8–40,2)	14,1 (11,1–17,8)
	*Parto vaginal* a	72,6 (69,0–75,8)	79,3 (73,0–84,4)	76,1 (63,7–85,2)	76,3 (71,2–80,7)	68,6 (56,1–78,9)	75,5 (71,0–79,5)
	Mulheres com parto vaginal^a^	329	255	140	657	67	724
	Enfermeiro/a/ obstetriz assistiu o parto	23,1 (10,6–43,3)	13,5 (5,1–31,3)	10,4 (2,0–39,4)	22,6 (13,0–36,2)	0,2 (0,0–1,3)	16,6 (8,6–29,5)
	Posição verticalizada	96,0 (93,2–97,7)	97,0 (94,0–98,5)	89,2 (79,9–94,5)	95,2 (92,0–97,1)	93,7 (72,6–98,8)	95,0 (92,0–97,0)
	Realização de Kristeller	6,9 (5,8–8,1)	7,9 (5,8–10,7)	4,6 (1,5–13,2)	7,2 (5,7–8,9)	3,3 (1,1–9,5)	6,8 (5,4–8,5)
	Realização de episiotomia	6,4 (5,1–8,1)	8,5 (3,5–19,2)	4,1 (0,5–25,0)	6,8 (4,0–11,3)	6,1 (2,4–14,6)	6,7 (4,1–10,8)

IC: intervalo de confiança.

a Inclui parto instrumental (fórceps ou com uso de vácuo extrator).

Em comparação com mulheres residentes na RM e/ou interior, as residentes no MRJ apresentavam maior proporção de mulheres com financiamento privado, de cor branca, com idade de 35 anos ou mais, com 16 ou mais anos de estudo, multíparas com PV anterior, início mais precoce e maior adequação do PN, bem como menor proporção de mulheres que peregrinaram (Tabela 1). Apresentavam menor proporção de risco gestacional, exceto infecções na admissão para o parto; maior proporção de TP; de PV e de cesariana intraparto; presença de acompanhante; maior uso de analgesia; e de partos na posição verticalizada e com atuação da EO (Tabela 2).

Comparadas às mulheres que tiveram seus partos em instituições com financiamento público, as que usaram os serviços privados são em maior proporção brancas, com idade ≥ 35 anos, maior escolaridade, vivem com companheiro, são nulíparas ou com cesariana anterior. Iniciam o PN cedo, têm maior adequação e menor peregrinação (Tabela 1). A maior proporção de nascimentos é por cesariana, principalmente anteparto, com acompanhante. Referem em menor proporção terem sido assistidas por EO/O no TP e parto. Também receberam, em maior proporção, as intervenções cateter venoso periférico e analgesia peridural, com 69% das mulheres que tiveram TP evoluindo para PV (Tabela 2).

As variáveis associadas ao desfecho "entrar em TP" foram: parto em hospital no MRJ e financiamento público (nível distal), e ser nulípara ou multípara com PV anterior (em comparação com multíparas com cesariana anterior), não ser obesa, ausência de risco obstétrico e ter preferência por PV no fim da gestação (nível intermediário) (Tabela 3).

**Tabela 3:** Trabalho de parto (TP) associado a características sociodemográficas, da atenção pré-natal e obstétricas, Pesquisa Nascer no Brasil II, estado do Rio de Janeiro (NBII-ERJ), 2021–2023.

		Teve TP (%)	Bruta		Ajustada	
			OR	IC	OR	IC
NÍVEL 1^a^		54,4				
*Sociodemográficas*						
Localização do Hospital						
	Município do Rio de Janeiro	63,6	2,2	(0,7–7,4)	3,7	(1,7–7,8)
	Região Metropolitana	51,1	1,3	(0,7–2,4)	1,4	(0,9–2,4)
	Outros Municípios	43,9	ref	-	ref	-
Financiamento do Parto						
	Público	66,0	7,2	(3,7–14,2)	9,7	(5,1–18,6)
	Privado	21,2	ref	-	ref	-
Cor da pele						
	Branca	47,1	ref	-	ref	-
	Preta	57,9	1,54	(1,1–2,2)	0,71	(0,5–1,1)
	Parda	57,5	1,52	(1,1–2,1)	1,03	(0,7–1,5)
Idade da mãe (anos)						
	10 a 19	71,8	3,3	(1,2–9,2)	1,66	(0,5–5,2)
	20 a 34	54,6	1,5	(1,0–2,3)	1,17	(0,8–1,8)
	35 ou mais	43,7	ref	-	ref	-
Escolaridade (anos)						
	≤ 8 (EF incompleto)	67,5	3,6	(2,4–5,6)	0,7	(0,3–1,6)
	9 a 11 (EF completo)	61,6	2,8	(1,9–4,1)	0,6	(0,3–1,2)
	12 a 15 (EM completo)	51,9	1,9	(1,3–2,7)	0,7	(0,4–1,2)
	≥ 16 (ES completo)	36,3	ref	-	ref	-
Vive com companheiro						
	Não	63,1	1,6	(1,23–1,96)	0,9	(0,7–1,2)
	Sim	52,4	ref	-	ref	-
NÍVEL 2^b^						
*História de cesariana anterior*						
	Nulípara	53,0 2,8		(2,1–3,6)	2,5	(1,8- 3,5)
	Multípara apenas com vaginal anterior	83,9	12,8	(8,5–19,1)	9,5	(5,3–17,4)
	Multípara com cesariana anterior	29,1	ref	-	ref	-
*Pref. tipo parto final gestação*						
	Vaginal	71,7	5,1	(3,8–6,8)	2,7	(1,9–3,7)
	Cesariana ou sem preferência	33,3	ref	-	ref	-
*Atenção pré-natal (PN)*						
Adequação do PN^c^						
	Não	64,4	1,8	(1,1–2,9)	1,1	(0,7–1,6)
	Sim	50,6	ref	-	ref	-
Peregrinação para o parto						
	Não	53,0	ref	-	ref	-
	Sim	65,1	1,7	(1,05–2,6)	1,5	(0,8–2,9)
*Obesidade pré-gestacional*						
	Não	58,1	2,0	(1,59–2,5)	1,5	(1,0–2,2)
	Sim	40,8	ref	-	ref	-
*Risco obstétrico* d						
	Não	61,1	2,4	(2,0–3,0)	3,8	(2,3- 6,4)
	Sim	39,3	ref	-	ref	-

OR: *odds ratio*. IC: intervalo de confiança de 95%. EF: ensino fundamental. EM: ensino médio. ES: ensino superior.

a Nível 1: ajustado pela localização do hospital e financiamento do parto.

b Nível 2: ajustado pela localização do hospital, financiamento do parto, história de cesariana anterior, preferência pelo tipo de parto no final da gestação, obesidade pré-gestacional e risco obstétrico.

c Início até 12 semanas de gestação e número de consultas adequado para a idade gestacional no nascimento segundo critério da OMS.

d Diabetes (crônica ou gestacional); síndromes hipertensivas (hipertensão arterial [HA] crônica, HA gestacional, pré-eclâmpsia, eclâmpsia, HELLP); doença crônica grave (lúpus, esclerodermia, doença renal, doença cardíaca, câncer); infecções no diagnóstico da internação; DPP; placenta acreta/increta/percreta; CIUR; gemelaridade; infecção pelo HIV; e internação durante a gestação.

As variáveis associadas ao PV foram ter menos de 15 anos de estudo e viver sem companheiro (nível distal); ser nulípara ou multípara apenas com PV anterior (nível intermediário), e presença de doula, poder se alimentar e se movimentar, uso de métodos não farmacológicos para alívio da dor, ser acompanhada no TP por EO/O) e uso de analgesia (nível proximal) (Tabela 4).

**Tabela 4:** Parto vaginal associado a características sociodemográficas, obstétricas e de atenção ao pré-natal, trabalho de parto e parto, Pesquisa Nascer no Brasil II, estado do Rio de Janeiro (NBII-ERJ), 2021–2023.

		Parto vaginal (%)	Bruta		Ajustada	
			OR	IC	OR	IC
NÍVEL 1^a^		75,5				
*Sociodemográficas*						
Localização do Hospital						
	Município do Rio de Janeiro	72,6	0,8	(0,5–1,6)	0,9	(0,5–1,9)
	Região Metropolitana	79,3	1,2	(0,6–2,5)	1,1	(0,5–2,5)
	Outros Municípios	76,1	ref	-	ref	
Financiamento do Parto						
	Público	76,3	1,5	(0,8–2,6)	0,6	(0,2–1,7)
	Privado	68,6	ref	-	ref	
Cor da pele						
	Branca	75,3	ref	-	ref	
	Preta	80,0	1,3	(0,8–2,3)	1,0	(0,6–1,7)
	Parda	74,0	0,9	(0,6–1,5)	0,8	(0,5–1,4)
Idade da mãe (anos)						
	10 a 19	82,0	1,4	(0,7–2,6)	1,3	(0,6–2,4)
	20 a 34	77,1	ref	-	ref	-
	35 ou mais	61,5	0,5	(0,3–0,9)	0,6	(0,3–1,0)
Escolaridade (anos)						
	≤ 11 (menos que EM completo)	75,1	2,6	(1,3–5,2)	2,6	(1,1–6,5)
	12 a 15 (EM Completo)	82,0	4,0	(1,8–8,9)	4,6	(1,6–13,4)
	≥ 16 (ES Completo)	53,5	ref	-	ref	-
Vive com companheiro						
	Não	84,5	2,0	(1,2–3,4)	1,9	(1,1–3,1)
	Sim	73,0	ref	-	ref	-
NIVEL 2^b^						
*História obstétrica*						
História de cesariana anterior						
	Nulípara	73,1	4,6	(2,8–7,4)	2,47	(1,5–4,0)
	Multípara apenas com vaginal anterior	91,5	18,0	(9,4–34,2)	13,28	(5, 6–31,6)
	Multípara com cesariana anterior	37,4	ref	-	ref	-
*Pref. tipo parto final gestação*						
	Vaginal	80,8	2,7	(1,4–5,1)	1,8	(0,91–3,7)
	Cesariana ou sem preferência	61,4	ref	-	ref	-
*Atenção pré-natal (PN)*						
Adequação do PN^c^						
	Não	76,6	1,1	(0,9–1,4)	0,7	(0,4–1,2)
	Sim	75,0	ref	-	ref	-
Peregrinação para o parto						
	Não	75,1	ref	-	ref	-
	Sim	78,1	1,2	(0,4–3,4)	1,4	(0,4–5,7)
*Obesidade pré-gestacional*						
	Não	77,8	1,5	(0,7–3,3)	1,2	(0,6–2,4)
	Sim	69,6	ref	-	ref	-
*Risco obstétrico* d						
	Não	74,1	ref	-	ref	-
	Sim	80,4	1,4	(1,0–2,1)	1,5	(0,9–2,4)
NIVEL 3^b^ *Boas práticas*						
Acompanhante no TP e parto						
	Não	66,2	ref	-	ref	-
	Sim	78,7	1,9	(0,8–4,3)	2,6	(1,0–7,0)
Presença de doula						
	Não	74,8	ref	-	ref	-
	Sim	89,6	2,9	(0,9–9,2)	4,3	(1,2–15,8)
Pôde se alimentar^e^						
	Não	68,8	ref	-	ref	-
	Sim	88,1	3,1	(2,2–4,3)	3,1	(2,2–4,6)
Movimentação^e^						
	Não	65,3	ref	-	ref	-
	Sim	85,8	3,7	(1,9–6,9)	3,46	(1,8–6,6)
Uso de métodos não farmacológicos^e^						
	Não	69,0	ref	-	ref	-
	Sim	84,0	2,8	(1,9–4,1)	3,3	(2,12–5,1)
TP acompanhado por enfermeira^e^						
	Não	62,6	ref	-	ref	-
	Sim	79,7	3,9	(1,1–14,2)	3,37	(1,1–10,5)
*Intervenções*						
Uso de acesso venoso periférico^e^						
	Não	74,9	ref	-	ref	-
	Sim	76,1	1,2	(0,7–2,3)	1,2	(0,5–2,9)
Uso de ocitocina^e^						
	Não	76,0	ref	-	ref	-
	Sim	72,6	0,8	(0,31–2,2)	0,6	(0,2–2,1)
Uso de analgesia^e^						
	Não	75,1	ref	-	ref	-
	Sim	84,6	2,3	(1,0–5,0)	5,8	(1,8–18,9)
Amniotomia^e^						
	Não	73,3	ref	-	ref	-
	Sim	88,6	2,2	(1,2–4,0)	1,8	(1,0–3,3)

OR: *odds ratio*. IC: intervalo de confiança de 95%. EF: ensino fundamental. EM: ensino médio. ES: ensino superior.

a Nível 1: ajustado pelo financiamento do parto, idade, nível de escolaridade e vive com companheiro.

b Níveis 2 e 3 ajustados pelo financiamento do parto, idade, nível de escolaridade, vive companheiro e história de cesariana anterior.

c Início até 12 semanas de gestação e número de consultas adequado para a idade gestacional no nascimento segundo critério da OMS.

d Diabetes (crônica ou gestacional); síndromes hipertensivas (hipertensão arterial [HA] crônica, HA gestacional, pré-eclâmpsia, eclâmpsia, HELLP); doença crônica grave (lúpus, esclerodermia, doença renal, doença cardíaca, câncer); infecções no diagnóstico da internação; DPP; placenta acreta/increta/percreta; CIUR; gemelaridade; infecção pelo HIV; e internação durante a gestação.

e Para a avaliação das boas práticas e intervenções, incluímos nas regressões brutas e ajustadas apenas as mulheres com tempo de trabalho de parto entre 2 e 16 horas (quintis 2, 3 e 4, descritos na Tabela 2).

## DISCUSSÃO

Há desigualdade na atenção ao parto no ERJ por área geográfica, tipo de financiamento e condição sociodemográfica das parturientes. O MRJ apresenta melhor perfil socioeconômico, maior proporção de TP e maior uso de algumas boas práticas recomendadas pelo MS8 e OMS7, tais como presença de acompanhante, assistência por EO/O e posições verticalizadas para o parto. Em municípios do interior e no setor privado, há maior ocorrência de cesarianas, principalmente aquelas realizadas antes do TP. No interior, observou-se maior frequência de mulheres com risco obstétrico, embora as parturientes com maior idade estejam no MRJ.

As boas práticas no TP/P estão disponíveis em todas as três áreas do ERJ, mas a presença de doula, acompanhante de livre escolha e ingestão de líquidos ou alimentos durante o TP foram mais frequentes em mulheres com financiamento privado do parto. Maior frequência de intervenções também foi observada nesse grupo de mulheres, bem como menor atuação das EO/O.

Entrar em TP mostrou associação com atendimento no MRJ, financiamento público do parto, ser nulípara ou multípara com PV anterior, ter preferência pelo PV no final da gestação e não ter obesidade ou intercorrências na gestação. Já o PV mostrou associação com menor escolaridade e ausência de companheiro, ser nulípara ou multípara com PV anterior, usar boas práticas no TP/P e analgesia, independentemente do tipo de financiamento e localização do hospital.

Os melhores indicadores observados no MRJ podem ser compreendidos como resultados de uma política de humanização da atenção ao parto que se iniciou em 1994, com a inauguração da maternidade Leila Diniz e posterior extensão desse novo modelo de atenção ao parto a todas as maternidades sob gestão da Secretaria Municipal de Saúde do RJ. A implantação do programa "Cegonha Carioca", em 2011^5^, reduziu a peregrinação e ampliou o conforto e a segurança das gestantes. Atualmente o MRJ é responsável por atender 90% dos partos do SUS na cidade.

Na RM, a criação de grandes maternidades estaduais na década de 2010 melhorou o acesso às boas práticas na atenção ao parto, aproximando-se do padrão do MRJ, exceto na presença de acompanhante no TP/P, embora essa prática se tenha expandido significativamente no ERJ. A lei que garante esse direito é de 2005^11^, mas no Brasil, em 2011, apenas 18% das parturientes contavam com a presença em tempo integral de um acompanhante da sua escolha^12^. Neste estudo, esse valor alcança 80% no MRJ/interior e 98% no setor privado.

As EO/O assistiram mais de 60% do TP das mulheres no ERJ, exceto no setor privado. No entanto, a assistência ao parto por EO/O foi de apenas 17%, sendo mais frequente no MRJ e nos serviços públicos. Isso indica espaço para aumentar a presença dessas profissionais nas maternidades do ERJ. Desde 1998, o MRJ coopera com a Faculdade de Enfermagem da Universidade Estadual do Rio de Janeiro (UERJ) para qualificar EO para a atenção PN e ao parto, e essas profissionais foram absorvidas pelas maternidades do ERJ^13^. A importância das EO/O no parto foi destacada em diversos estudos, incluindo um artigo da revista *Lancet*, que ressaltou seu papel em otimizar a fisiologia do parto, reduzir intervenções e melhorar desfechos^14^. Nove et al.^15^ estimaram que mais de 60% das mortes maternas, neonatais e fetais em países de baixa renda poderiam ser evitadas com a presença dessas profissionais^16^.

O uso de ocitocina para acelerar o TP e a amniotomia reduziram-se à metade em relação aos dados nacionais do NB, primeiro inquérito perinatal brasileiro realizado em 2011, enquanto a manobra de Kristeller e episiotomia reduziram 80%^9^. A litotomia relatada por 92% das brasileiras no NB^9^ foi substituída no ERJ pela posição verticalizada para parir (95%), sem distinção por área ou tipo de financiamento. A posição verticalizada (sentada, recostada, de cócoras, ou de pé) é recomendada para abreviar o período expulsivo e reduzir a episiotomia e o uso de fórceps^17,18^.

Todas essas mudanças sofreram também a influência do programa "Rede Cegonha", desenvolvido pelo governo federal a partir de 2011, com o objetivo central de humanização do atendimento ao parto nas maternidades do SUS^19,20^. Ainda, não pode ser esquecido o movimento organizado das mulheres, principalmente na classe média informada. A luta delas pelo respeito à fisiologia, pelo direito à escolha do tipo de parto e pela autonomia na condução do nascimento do seu filho influenciou os serviços privados a mudarem condutas e adotarem as práticas com recomendação científica^21,22^.

No setor privado, constata-se que quando a parturiente entra em TP evolui para PV quase tanto quanto no setor público. Contudo, o modelo da cesariana anteparto é generalizado e uma prática difícil de mudar, mesmo com iniciativas como o Parto Adequado^23^, que vêm se dedicando à valorização do parto normal e à redução do percentual de cesarianas desnecessárias na saúde suplementar. Uma das consequências das cesarianas anteparto é o excesso de nascimentos com 37–38 semanas gestacionais, chamados termo precoce. O NB mostrou que quase metade dos bebês do setor privado nascia a termo precoce, e neles eram mais frequentes resultados adversos como taquipneia transitória, internação em unidade de terapia intensiva neonatal, icterícia e infecções^24^.

A presença da doula durante o TP/parto, mais frequente no MRJ e no setor privado, oferece apoio contínuo, incentivo, facilita a comunicação da mulher com a equipe de saúde e contribuiu neste estudo para a ocorrência do PV^25^. A presença do acompanhante de escolha da mulher, embora tenha perdido significância após o ajuste para outras variáveis, também é uma importante medida para aumentar o suporte social no parto, bem como resgatar o nascimento como um evento familiar^12^.

Ser atendida em serviço público e ter um PV anterior foram as principais variáveis que contribuíram para o desfecho TP no ERJ. A preferência da mulher pelo PV no fim da gestação também foi um fator importante para ela entrar em TP. Estudos sobre o tema mostram a relevância de apoiar e promover o PV durante a assistência PN^26^. Ressalta-se que a preferência da mulher não mostrou associação com o PV, mostrando que outras questões, como o modelo de atenção com o uso de boas práticas, ganham maior relevância para o desfecho parto.

Neste estudo, o uso de boas práticas no TP aumentou a chance de as mulheres terem PV, resultado coerente com a literatura sobre o tema. A maioria dos estudos sobre os efeitos das boas práticas obstétricas conduzidos em países desenvolvidos também se aplica à realidade brasileira^7^. O PV mostrou associação com a história obstétrica (nuliparidade ou antecedente de parto vaginal), sendo um resultado relevante, já que uma das principais estratégias para a redução das cesarianas no Brasil é a prevenção da primeira cesariana, evitando as cesarianas de repetição. Por fim, a analgesia no TP também mostrou associação com o PV, sendo uma tecnologia útil para o alívio da dor do parto, podendo ser uma aliada na redução de cesarianas^27^. A oferta de analgesia peridural traz conforto para a parturiente e deve ser ampliada, especialmente no SUS, onde é pouco ofertada. O medo da dor é a principal razão para o desejo de cesariana^26^, e avanços nas técnicas e nos fármacos têm tornado a analgesia cada vez mais segura para mãe e bebê^27,28^.

Pontos fortes desta pesquisa são o desenho do estudo, que viabilizou a representação de todas as grandes áreas geográficas do ERJ, e tipo de financiamento de parto; o número representativo de mulheres; e a baixa taxa de recusa (dados não mostrados), contribuindo para a robustez do estudo. Como limitações, os resultados não se aplicam a hospitais com menos de cem partos/ano; o financiamento público inclui partos em hospitais públicos e privados conveniados ao SUS, e a análise não fez distinção entre esses dois tipos de serviço; e variáveis relativas às complicações são provenientes de prontuários médicos, dependendo da qualidade do registro em cada hospital. A ausência de registro foi considerada ausência da complicação. Estudos que validem a ausência de registros nos prontuários como ausência de complicações e que aprimorem a aferição do início e da duração do TP são essenciais para pesquisas futuras.

Conclui-se que ocorreram avanços na assistência ao parto no ERJ, com destaque para o MRJ, embora a frequência de TP e de PV continue baixa. A adoção das boas práticas se associou positivamente a ter PV, com maior magnitude perante a atuação de doulas e o uso de analgesia. Mulheres em condições de vulnerabilidade social tiveram maior chance de PV. Os resultados deste estudo apontam para as principais práticas promotoras do PV no ERJ e podem subsidiar a melhoria da assistência.

## Data Availability

Os conjuntos de dados gerados e/ou analisados durante o estudo estão disponíveis com o autor correspondente mediante solicitação.
